# Chromosome Fusion Affects Genetic Diversity and Evolutionary Turnover of Functional Loci but Consistently Depends on Chromosome Size

**DOI:** 10.1093/molbev/msab185

**Published:** 2021-06-19

**Authors:** Francesco Cicconardi, James J Lewis, Simon H Martin, Robert D Reed, Charles G Danko, Stephen H Montgomery

**Affiliations:** 1 School of Biological Sciences, University of Bristol Bristol—Life Sciences Building, Bristol, United Kingdom; 2 Department of Zoology, University of Cambridge, Cambridge, United Kingdom; 3 Baker Institute for Animal Health, Cornell University, Ithaca, NY, USA; 4 Ecology and Evolutionary Biology, Cornell University, Ithaca, NY, USA; 5 Institute of Evolutionary Biology, University of Edinburgh, Edinburgh, United Kingdom

**Keywords:** chromosome fusion, chromosome size variation, repetitive elements, intron size, recombination rate, genome architecture, genome evolution

## Abstract

Major changes in chromosome number and structure are linked to a series of evolutionary phenomena, including intrinsic barriers to gene flow or suppression of recombination due to chromosomal rearrangements. However, chromosome rearrangements can also affect the fundamental dynamics of molecular evolution within populations by changing relationships between linked loci and altering rates of recombination. Here, we build chromosome-level assembly *Eueides isabella* and, together with a recent chromosome-level assembly of *Dryas iulia*, examine the evolutionary consequences of multiple chromosome fusions in *Heliconius* butterflies. These assemblies pinpoint fusion points on 10 of the 20 autosomal chromosomes and reveal striking differences in the characteristics of fused and unfused chromosomes. The ten smallest autosomes in *D. iulia* and *E. isabella*, which have each fused to a longer chromosome in *Heliconius*, have higher repeat and GC content, and longer introns than predicted by their chromosome length. When fused, these characteristics change to become more in line with chromosome length. The fusions also led to reduced diversity, which likely reflects increased background selection and selection against introgression between diverging populations, following a reduction in per-base recombination rate. We further show that chromosome size and fusion impact turnover rates of functional loci at a macroevolutionary scale. Together these results provide further evidence that chromosome fusion in *Heliconius* likely had dramatic effects on population level processes shaping rates of neutral and adaptive divergence. These effects may have impacted patterns of diversification in *Heliconius*, a classic example of an adaptive radiation.

## Introduction

Structural changes in the genome can be an important factor for speciation (Feulner and De-Kayne 2017), population divergence, and adaptation (De Storme and Mason 2014). Although studies of structural evolution often focus on small to medium-scale structural variants, such as inversions or translocations, large-scale structural changes, like chromosome fusion, can have substantial and immediate evolutionary impacts. The consequences of chromosome fusions occur via two main effects. First, fusions can disrupt meiotic sorting of chromosomes in heterozygous individuals or result in imbalanced gametes, both of which can lead to reproductively isolated “chromosomal races” (Dobzhansky and White 1977; Hauffe and Searle 1998; de Vos et al. 2020). Second, fusion events reduce the number of unlinked DNA molecules, which results in less independence among loci. This lower per chromosome recombination rate may be favored in certain circumstances. For example, fusions may aid adaptation if recombination is reduced between coadapted alleles at multiple loci (Guerrero and Kirkpatrick 2014; Veller et al. 2019, 2020). A beneficial alteration of the recombination landscape might explain why chromosomal fusions could become fixed despite initial deleterious impacts on meiosis in species with monocentric chromosomes (Lunt et al. 2014; Fradin et al. 2017).

The altered recombination rate associated with chromosome fusions can have permanent downstream consequences, affecting both the efficacy of purifying selection (Charlesworth 1990) and the impact of selection on linked sites, which consequently determine levels of genetic diversity (Cutter and Payseur 2013; Corbett-Detig et al. 2015). Indeed, although the effect is modest, genome-wide nucleotide diversity in 38 butterfly species was found to be correlated with chromosome number (Mackintosh et al. 2019). Although neutral DNA is highly susceptible to nonselective evolutionary processes such as variation in recombination rate, functional DNA elements (e.g., genes, enhancers, promoters, etc.) may be more constrained due to selective forces. Turnover of functional DNA (e.g., gain or loss of conserved *cis*-regulatory elements [CREs]) is strongly associated with divergence time between taxa (Villar et al. 2015; Lewis et al. 2016; Lewis and Reed 2019), but also mechanisms and processes that implicate selection, such as epistasis, gene pleiotropy, adaptive population divergence, and positive selection from environmental pressures (Kim et al. 2016; Van Belleghem et al. 2017; Concha et al. 2019; Lewis et al. 2019, 2020; Moest et al. 2020). Thus, the extent to which changes in recombination rate might alter functional DNA evolution remains relatively unknown, and prior studies seemingly support the potential for both strong and weak effects.

In *Heliconius* butterflies, 10 pairs of the 31 ancestral chromosomes are thought to have fused, resulting in 21 chromosomes, whereas the ancestral chromosome number is retained in their sister genus *Eueides* and most other outgroup genera within Heliconiini (Davey et al. 2016). The impact of these chromosome fusions on the recombination landscape may be especially pronounced in *Heliconius* due to their holocentric chromosomes where multiple kinetochores, rather than a single centromere, may favor the escape of some of the deleterious effects of chromosome fusions (Mandrioli and Manicardi 2020). There also appears to be a strict rule of one crossover per chromosome per meiosis in this clade (Davey et al. 2016). Fused chromosomes should therefore have a reduced recombination rate per base pair (bp) compared with their ancestral, unfused progenitors. In *H. melpomene*, genetic diversity is strongly and negatively correlated with chromosome length (Martin et al. 2016), and longer chromosomes show reduced levels of introgression between *Heliconius* species (Edelman et al. 2019; Martin et al. 2019). Therefore, the fusion events that produced the ten longest chromosomes in *Heliconiu*s likely altered the evolutionary trajectories of the ten pairs of shorter progenitor chromosomes involved. Indeed, Davey et al. (2016) hypothesized that the altered genome structure may contribute to the elevated speciation rate in *Heliconius* (Kozak et al. 2015). Here, we aimed to test whether increases in chromosome length have altered levels of nucleotide diversity and rates of evolutionary turnover in *Heliconius*. We combine our newly available *Dryas iulia* genome assembly (Lewis et al. 2021), with a chromosome-level assembly of *Eueides isabella*. We then compare these genomes, which have the ancestral chromosome number of 31, with the genomes of *H. melpomene* and *H. erato*. Our findings show that the ten smallest chromosomes in *Dryas* and *Euiedes*, which are fused in *Heliconius*, have a distinct genomic and gene-structural composition. Chromosome fusions are associated with a dramatic change in recombination and background selection. Finally, we show that at a macroevolutionary time scale this had a significant effect on DNA content putatively under selection.

## Results and Discussion

### Chromosome-Level Assembly of the *Eueides isabella* Genome and Serial Chromosome Fusion at the Origin of *Heliconius*

Using a combination of high coverage long (Pacific Biosciences reads) and short-read data, we generated a highly complete de novo assembly for *E. isabella* (supplementary fig. S1, Supplementary Material online). Final chromosome-level scaffolding brought the contiguity to 14.7 Mb with a final genome size of ∼440 Mb, and very minimal N/X characters (<55 kb) (supplementary figs. S1−S4, Supplementary Material online). Genome sequence composition was highly similar between *E. isabella*, *D. iulia*, and the *H. melpomene* and *H. erato* reference genomes (supplementary table S1, Supplementary Material online). Genome completeness, as measured using the BUSCO database (insect_db9: 1658 gene), was high, with 99.3%, of genes being complete (single + duplicated) and only 0.5% (9/1658) of genes missing. Again, extremely similar results were found for *D. iulia* and the two *Heliconius* genomes (supplementary table S1 and fig. S5, Supplementary Material online). Therefore, these species represent four of the highest quality lepidopteran genomes.

**Fig. 1.**
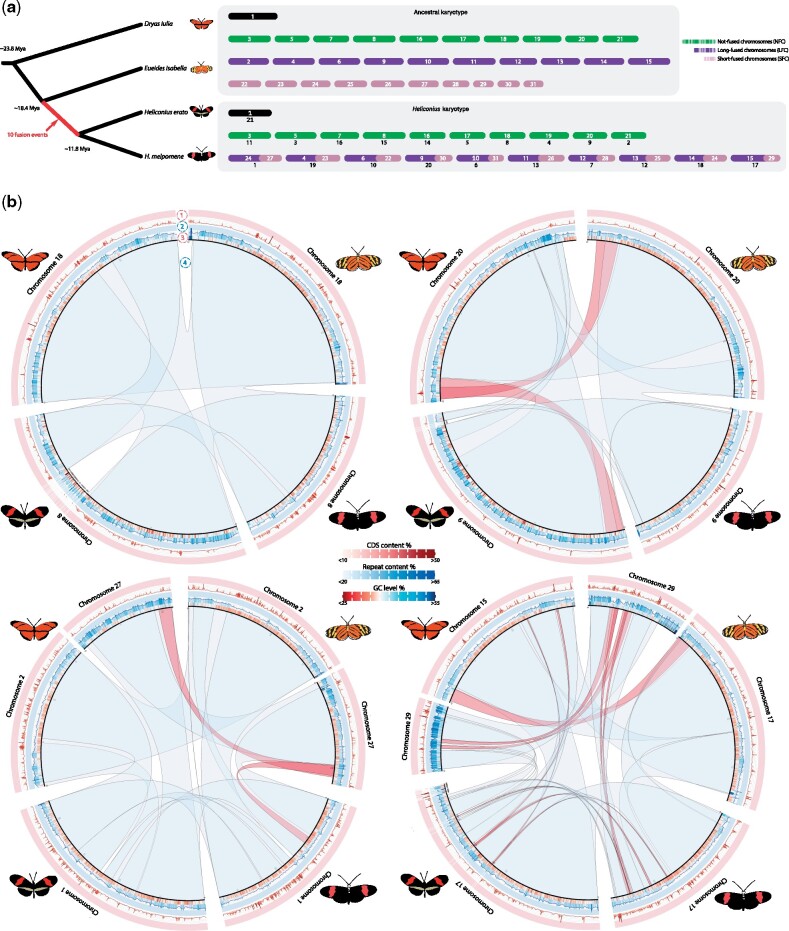
Synteny plots for four representative chromosome fusions. The comparisons are shown only for the flanking species, in order to avoid confusion. For layer of for the plots indicate: 1) coding region content, higher density is shown in darker red; 2) repetitive element richness, higher density is shown in darker blue; 3) GC level, in red AT-rich regions while in blue more GC-rich content; 4) ribbons showing synteny blocks (light blue), blocks are red colored when the block is inverted in the comparison. The ideogram scale is in Mbp.

Our *E. isabella* assembly recovered the 31 expected chromosomes in 31 contiguous sequences. *E. isabella* chromosomes displayed a highly conserved synteny with *D. iulia* and few putative inversions (mostly at the end of chromosomes) or translocations (fig. 1; supplementary figs. S6−S8, Supplementary Material online). This allowed us to identify the ancestral-chromosome fusion points in the two *Heliconius* species with a high degree of accuracy, all of which were close to their previously estimated locations (Davey et al. 2016). We also combined multiple annotation methods to predict 26,555 protein-coding genes in the *E. isabella* genome (supplementary table S1, Supplementary Material online). This estimate is slightly higher than the other Heliconiini genomes, probably due to a higher degree of gene model fission as indicated by the lower distribution of completeness (supplementary fig. S9, Supplementary Material online). Repetitive elements account for 29.80% of the *E. isabella* genome, which is slightly less than in *D. iulia* and *H. erato* (31.74% and 33.02%, respectively), but significantly more than *H. melpomene* (25.90%; supplementary table S1 and fig. S10, Supplementary Material online) and, in line with other Heliconiini genomes, total interspersed repeats constitute the largest fraction of repetitive elements (23.91%).

### Chromosome Fusion Leads to a Change in the Spatial Distribution of Repetitive Elements

**Fig. 2.**
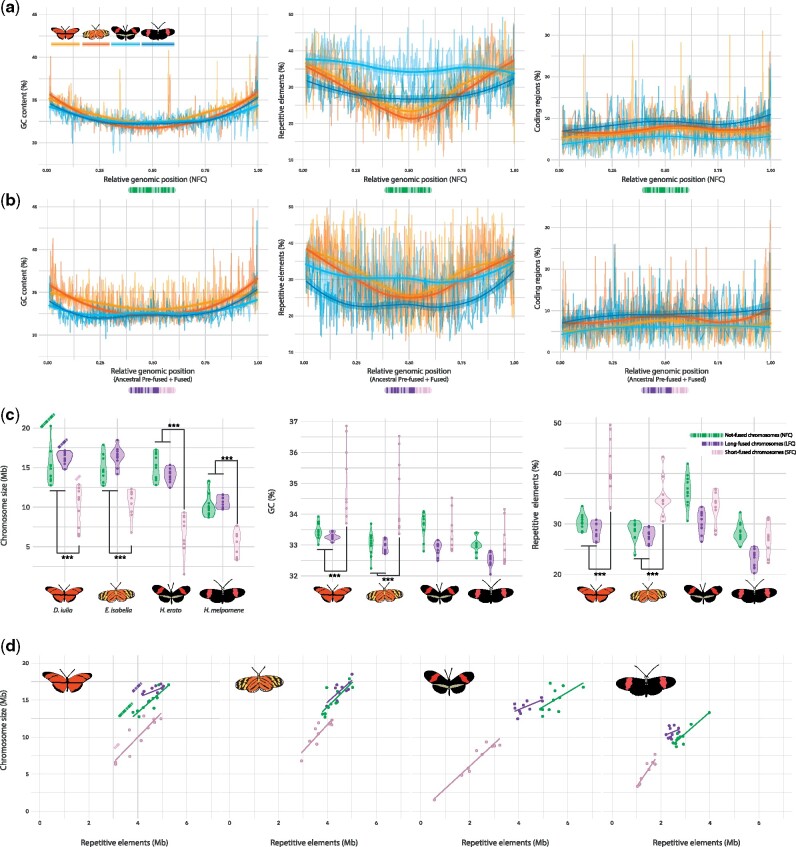
(*a*) GC content (%), repeat content (%) and coding density within 100 kb nonoverlapping windows in not fused chromosomes (NFC) of the four species (showed in color). (*b*) Same as (*a*) but for *Heliconius* fused chromosomes and their homologous in *Dryas iulia* and *Eueides isabella*. In both cases, GC and repeat distributions are accumulating at the chromosome tails, whereas coding density seems to be slightly denser in the central part of chromosomes. For fused chromosomes in *Heliconius* spp. fusion points is placed at 0.5 of the *x*-axis. (*c*) Violin plots of the different chromosome types, showing how and where size, CG content (%) and repetitive elements (%) are different. Short-fused chromosomes (SFC) appear to be smaller and with higher GC and repeat content compared with not-fused (NFC) and long-fused (LFC) chromosomes, which, in turn, to not appear significantly different. ***Wilcoxon rank-sum test “less”; *P* values <0.001. (*d*) Scatter plots between chromosome size and their repetitive element (Mb), their relative regression line, for the different chromosome types (color) for each species.

Comparative analyses of chromosome composition in Lepidoptera has shown that the distribution of repeats, GC content, and gene content do not follow a consistent pattern across chromosome length (Ahola et al. 2014). We explored these features in our assemblies to test for an effect of chromosome fusions on chromosome composition. We found that across all four species, chromosome lengths are highly correlated with repeat content (*P *<* *0.0001; Pearson’s *ρ *= 0.89; supplementary fig. S11, Supplementary Material online). All chromosomes in *D. iulia*, *E. isabella*, and the unfused chromosomes in *Heliconius* have higher GC and repetitive element frequencies toward the chromosome ends. In contrast, coding region density shows little variation across the unfused chromosomes, with a slight increase in coding sequence frequency around chromosome centers (fig. 2*a*). This pattern is maintained among fused *Heliconius* chromosomes and their unfused homologous chromosomes in *D. iulia* and *E. isabella*, although the increase is mostly absent in *Heliconius* spp. (fig. 2*b*). If CG-rich and repetitive element accumulations in the chromosome’s tails were retained after chromosome fusions, then fusion events should produce a W-shaped distribution with peaks centered around the fusion point that reflect the remnants of the ancestral chromosomes. Although a small peak is present at the fusion point (0.5 of the *x*-axis), this increase is substantially lower compared with the tails (fig. 2*b*). This suggests selection acted to remove repetitive elements in the central part of chromosomes following the fusion events, whereas the difference in magnitude between *Heliconius* spp. and non-*Heliconius* spp. is likely due to the overall loss of repetitive elements in *Heliconius* spp., an observation previously described by Ray et al. (2019). Comparing the ten smallest Heliconiini chromosomes with their homologues in *Melitaea cinxia*, it seems clear that chromosome lengths have been highly stable over this long evolutionary time period. Our data therefore seem to contradict the notion that holocentric chromosomes have uniform distributions of GC, repeats and gene content across chromosomes (Grbić et al. 2011; Mandrioli and Manicardi 2020), and contrasts with monocentric chromosomes where these features are compartmentalized to GC-rich and GC-poor regions (Bernardi et al. 1985). Whether the uneven distributions we observe are favored due to meiotic pairing or some other selective driver remains to be tested.

### Chromosomes That Were Fused in *Heliconius* Are Small and Have Distinct Nucleotide Compositions

Previous comparisons between *Bombyx mori, M. cinxia*, and *H. melpomene* suggested that lepidopteran chromosomal fusion events involved the shortest chromosomes (Ahola et al. 2014). These tend to have high rates of chromosomal evolution and rearrangement, and high proportions of repetitive elements (see above). These factors might explain the disproportionate involvement of short chromosomes in fusions in *Heliconius* (Ahola et al. 2014). We used our chromosome-level assemblies of *E. isabella* and *D. iulia* to test whether this pattern is consistent in a shallower phylogenetic framework, and to identify how chromosome fusion events have shaped *Heliconius* genomes.

To test the effects of chromosome fusions on *Heliconius* genomes, we split the fused chromosomes in *Heliconius* into their corresponding homologous chromosomes in *E. isabella* and *D. iulia*. These were categorized as: 1) not-fused chromosomes (NFC), homologous chromosomes that are compositionally conserved within Heliconiini; 2) long-fused chromosomes (LFC), the longer split chromosome sequence in *Heliconius* and its ancestral homolog; and 3) short-fused chromosomes (SFC), the shorter split chromosome sequence in *Heliconius* and its ancestral homolog. Across all species, SFCs are the smallest chromosomes (Wilcoxon rank-sum test “less”; *P *≤* *4.5 × 10^−08^), even in *D. iulia* and *E. isabella* (Wilcoxon *P *<* *0.01). *Heliconius melpomene* has smaller chromosomes overall (Mandrioli and Manicardi 2020) compared with the two outgroups (Wilcoxon *P *≤* *1.3 × 10^−11^), but in *H. erato* only the fused chromosomes, both LFC and SFC, differ in size from *D. iulia* and *E. isabella* (fig. 2*c* and *d*; Wilcoxon *P *≤* *0.00016). This indicates that *H. melpomene* experienced a secondary reduction in genome size after the fusion events, with further reductions in chromosome size occurring in the unfused chromosomes, whereas *H. erato* did not (supplementary fig. S13, Supplementary Material online).

Within *D. iulia* and *E. isabella*, SFCs (which are not fused in these species) displayed a different composition from other chromosomes, with significantly greater GC and repetitive element content in SFCs (Wilcoxon rank-sum test “greater”; *P *≤* *6.7 × 10^−06^) (fig. 2*c*). The relationship between chromosome size and repetitive elements across species and chromosome types (NFC, LFC, and SFC) also shows striking differences. Our analysis revealed significant shifts in the distribution of the four different types of repetitive elements. Retroelements and total interspersed repeats seem to be significantly reduced in *Heliconius* compared with *E. isabella* and *D. iulia*, mainly in SFCs; whereas rolling circles and DNA transposons seem to be reduced specifically in *E. isabella* (supplementary fig. S11, Supplementary Material online). This indicates that rates of repetitive element expansion/contraction vary predictably between chromosome types. SFCs show particularly high interspecific variation in chromosome composition, with *D. iulia* having the highest repeat density, as reflected having the smallest chromosomes for a given repeat content (chr. size ∼ repetitive content, intercept = 0.23 ± 0.11; vs. other Heliconiini: *P*-adjusted ≤ 0.0018), followed by *E. isabella* and *H. erato* (0.31 ± 0.10 and 0.39 ± 0.07, respectively), and finally *H. melpomene* (0.53 ± 0.04; *P*-adjusted ≤ 8.2 × 10^−06^) (fig. 2*d*;supplementary table S2, Supplementary Material online). Hence, the more typical composition of SFCs in *Heliconius* is explained by SFCs shifting in composition following the fusion events to match those of the larger LFC and NFC chromosomes.

As recently reported in millipedes (Qu et al. 2020), genomes with more repetitive elements show significant expansions in intron size. We explored this effect in *Heliconius* by looking at the composition of annotated gene models in SFCs. Although transcript, CDS, exon, and UTR lengths show highly similar distributions across species, intron lengths differ between both species and chromosome types. *Dryas iulia* has significantly longer intron sizes (Wilcoxon rank-sum test “greater”; *P *<* *2.2 × 10^−16^; mean: 2,688 bp), followed by *E. isabella* (Wilcoxon rank-sum test “greater”; *P *<* *2.2 × 10^−16^; mean: 2,335 bp)*, H. erato* (Wilcoxon rank-sum test “greater”; *P *<* *2.2 × 10^−16^; mean: 1,770 bp), and *H. melpomene* (mean: 972 bp) (supplementary fig. S15, Supplementary Material online). We also found that, within species, intron length for genes on SFCs are significantly longer than for LFCs or NFCs (Wilcoxon rank-sum test “greater”; *P *≤* *2.63 × 10^−14^) (supplementary fig. S15, Supplementary Material online). This pattern is highly similar to that of repeat content, supporting evidence of a correlation between repeat content and intron length (Qu et al. 2020). Indeed, SFC introns contain significantly higher amounts of transposable elements (TE) than would be predicted by their length (*P *≤* *6.7 × 10^−09^; intercept for intergenic regions 0.79 ± 0.03; intercept for intronic regions 0.67 ± 0.02). In contrast, repeat content for introns and intergenic regions was not significantly different in NFCs and LFCs. For *D. iulia* and *E. isabella*, the slope of the relationship between repeat content and introns or intergenic regions showed no significant difference (*P *≥* *0.15). The *y*-intercept (elevation along the *y*-axis), however, did indicate a significant change, with intronic intercept ∼50% lower than intergenic regions (*P *<* *2.2 × 10^−16^; supplementary table S4, Supplementary Material online), reflecting the higher amount of repetitive elements in *Dryas*. Overall, these findings show how repetitive element content influence not only genome size but also the gene structure itself. Why introns are more affected by repetitive elements is not clear. It is possible that purifying selection may be stronger on intergenic regions, perhaps to avoid the disruption of regulatory elements in relatively compact genomes. Although regulatory elements do occur in introns, they are substantially less common in our ATAC-Seq data set (1 per 7,924 bp of intronic sequences, compared with 1 per 2,861bp of intergenic sequence). Alternatively, the presence of TE in intronic regions may favor exonization, the acquisition of new exons from nonprotein-coding, primarily intronic, DNA sequences (Sela et al. 2010; Schmitz and Brosius 2011).

### Chromosome Fusions Caused Reductions in Recombination Rate and Levels of Diversity

Although the fused chromosomes in *Heliconius* tend to be much longer than the unfused chromosomes (by ∼40–60% in *H. erato* and *H. melpomene*, respectively), genetic linkage maps (Davey et al. 2016; Van Belleghem et al. 2017) reveal that map lengths are consistently close to 50 centimorgan (cM) for all chromosomes, with fused chromosome maps only marginally longer than unfused (by 5% in *H. melpomene* and 7% in *H. erato*) (supplementary fig. S18, Supplementary Material online). This implies an average of one crossover per pair of bivalents per meiosis, regardless of chromosome length (Davey et al. 2016). If this trend was present in the 31 ancestral chromosomes, this would imply that fused chromosomes, especially SFCs, have experienced a dramatic decrease in their per-base recombination rate. For example, ancestral chromosome 31, an SFC forming part of chromosome 6 in *Heliconius*, accounts for just 6 cM of the total 48 cM (supplementary fig. S18, Supplementary Material online). We therefore hypothesized that the decrease in recombination rate would result in a decrease in genetic diversity for the SFCs and LFSc, as recombination rate determines the extent to which the processes of background selection and genetic hitchhiking tend to reduce diversity at linked sites (Cutter and Payseur 2013; Campos and Charlesworth 2019).

As a proxy for the level of neutral genetic diversity in each of the four species, we computed nucleotide diversity (π) at 4-fold degenerate third codon positions (hereafter π_4D_) using resequenced individuals (see supplementary table S5, Supplementary Material online for sample information and accession numbers). There is a strong negative relationship between chromosome length and π_4D_ in *D. iulia* (*R*^2^ = 0.506, *P *=* *1 × 10^−5^), *H. melpomene* (*R*^2^ = 0.752, *P *=* *1 × 10^−6^) and *H. erato* (*R*^2^ = 0.894, *P *=* *3 × 10^−10^) (supplementary fig. S19, Supplementary Material online), consistent with lower per-base recombination rates on longer chromosomes resulting in greater levels of background selection and/or hitchhiking (Martin et al. 2016). This trend was not seen in *E. isabella*, which generally shows low genetic diversity across all chromosomes. It is possible that a reduction in effective population size has reduced the efficacy of background selection in *E. isabella* such that chromosome-level recombination rate is no longer a good predictor of diversity. We therefore focused on *D. iulia* as representative of the ancestral state for comparison with the *Heliconius* species.

**Fig. 3.**
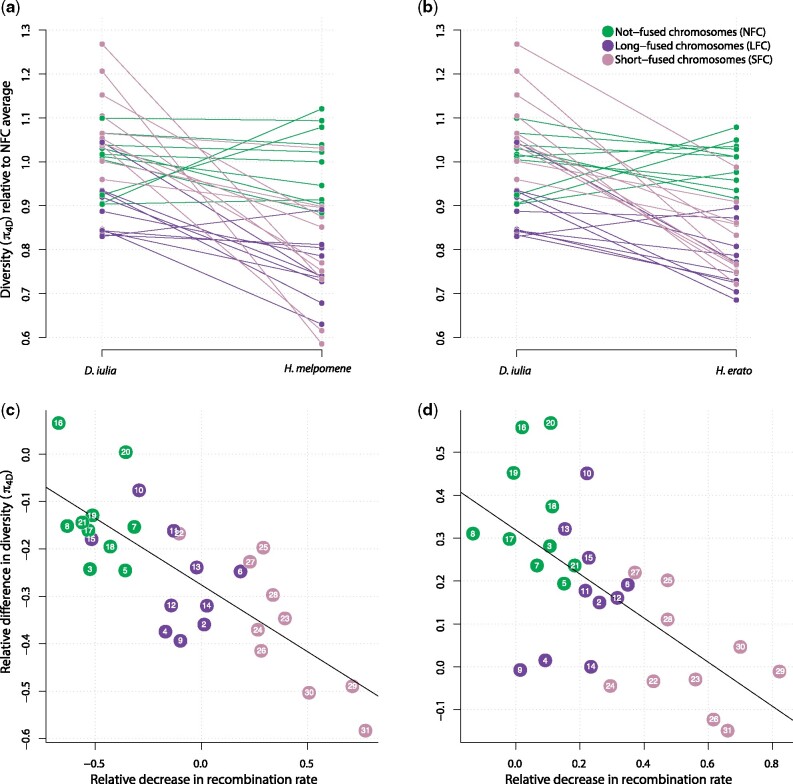
Loss of genetic diversity following chromosome fusions in *Heliconius*. (*a*, *b*) Nucleotide diversity at 4-fold degenerate codon positions (*π*_4D_) averaged across each of the 31 ancestral chromosomes, and normalized relative to the average *π*_4D_ for not-fused chromosomes (NFC) to account for different effective population sizes between species. The outgroup species *Dryas Iulia* is compared with *Heliconius melpomene* (*a*) and *H. erato* (*b*). Downward sloping lines indicate chromosomes that have lower relative genetic diversity in *Heliconius*. (*c*, *d*) The relative change in π_4D_ between the inferred ancestor and the extant species *H. melpomene* (*c*) and *H. erato* (*d*) plotted as a function of the relative decrease in recombination rate following the ten fusion events. The ancestral relative change in diversity and relative decrease in recombination rate are calculated assuming that the ancestral chromosomes had lengths and levels of diversity equal to those in *D. iulia*, and recombination map lengths of 50 cM each (see main text). Points further to the right indicate chromosomes that have experienced a greater decrease in recombination rate. Note that in *H. melpomene* the NFCs show negative values on the *x*-axis, indicating an overall increase in recombination rate, due to the genome-size reduction in *H. melpomene*. Nonetheless, the trend that fused chromosomes (especially SFCs) have experienced the greatest decrease in relative recombination rate and relative diversity is present in both *H. melpomene* and *H. erato*. A fitted linear regression line is included for convenience.

Relative levels of diversity tend to be reduced on both the LFCs and SFCs in *Heliconius* compared with their unfused homologues in *D. iulia* (fig. 3*a* and *b*). The difference is most pronounced for the SFCs, which tend to have experienced the greatest decrease in recombination rate following the fusion events. Indeed, assuming map lengths of 50 cM for *D. iulia* chromosomes, we can estimate recombination rates for each chromosome before and after the fusions. This estimated reduction in recombination rate is a strong predictor of the reduction in relative levels of diversity (Spearman’s *ρ* = 0.757, *P *=* *3 × 10^−6^ for *H. melpomene*; *ρ* = 0.662, *P *=* *1 × 10^−4^ for *H. erato*; fig. 3*c* and *d*).

Our findings demonstrate that the ten fusion events in the ancestor of all *Heliconius* species resulted in a dramatic change in the fate of the chromosomes involved. First, the reduced effective population size caused by increased background selection likely also leads to a reduction in the efficacy of selection. Adaptive evolution may be further impeded by Hill−Robertson interference between tightly linked selected loci (Hill and Robertson 1966; Barton 1995). Evidence for less efficient selection in genomic regions of reduced recombination rate has been reported in *Drosophila* (Presgraves 2005; Haddrill et al. 2007; MacKay et al. 2012). Given that the gene complement of each fused chromosome in *Heliconius* is largely unchanged from that in outgroup species, we hypothesize that the fusions may have resulted in a systematic shift in the relative importance of certain genes for adaptation. A second side-effect of reduced recombination rate in the fused *Heliconius* chromosomes is an increase in barriers against introgression between hybridizing species (Edelman et al. 2019; Martin et al. 2019). This occurs because introgressed tracts take longer to break down in regions of lower recombination rate and are consequently more rapidly purged by selection. This raises the intriguing possibility that the fusion events caused some regions of the genome to become less permeable to gene flow (Davey et al. 2016).

Combined with our assessment of TE abundance, our findings contrast with the widespread trend in other organisms for repetitive elements to be more common in regions of low recombination rate (reviewed by Kent et al. 2017). Instead, the ten smallest lepidopteran chromosomes have the highest recombination rates and the highest repeat content, and their repeat content appears to have decreased following the fusions events in *Heliconius* that lowered their recombination rates. It is generally thought that more efficient selection in regions of higher recombination rate should limit the spread of TEs. We therefore hypothesize that the efficiency of selection is not the only factor at play. For example, the rate of TE insertion may be greater on these small chromosomes, outweighing the effect of more efficient selection against them.

### Chromosome Fusions Alter the Rate of Turnover in Functional DNA

**Fig. 4.**
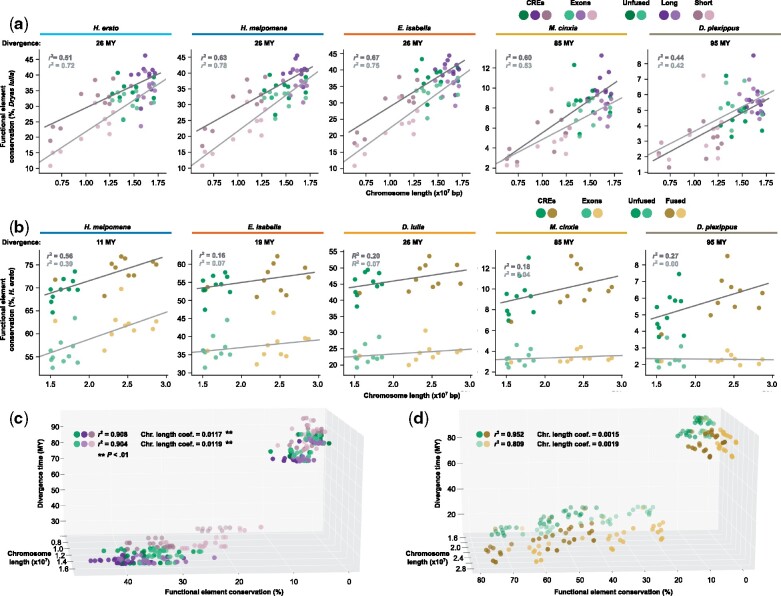
Patterns of functional divergence in fused and unfused chromosomes. (*a*) Percent of *Dryas iulia* CREs (dark circles) and gene exons (light circles) conserved over 95 million years of divergence (divergence times shown above individual plots). Long (purple), short (brown), and unfused (green) chromosomes all consistently show a strong correlation of DNA conservation with chromosome length. (*b*) Percent of *Heliconius erato* CREs (dark circles) and gene exons (light circles) conserved over 95 million years of divergence. Chromosome length is highly correlated with functional DNA conservation between *H. erato* and *H. melpomene*, both of which have 21 chromosomes. This correlation declines rapidly with divergence time and comparison with unfused chromosomes. Multiple regression analysis of chromosome length and divergence time on functional DNA conservations shows that chromosome length is significantly more impactful on *D. iulia* sequences (*c*) than for *H. erato* CRES or gene exons (*d*).

Finally, we sought to assess how the ten chromosome fusions in *Heliconius* have affected loci putatively under selection. Although it is clear that chromosome fusions can drive a decrease in neutral nucleotide diversity, the extent to which the recombination landscape alters the evolution of functional elements in the genome remains uncertain. Functional elements, such as protein coding sequences and CREs, likely face opposing forces from neutral processes driven by reduced recombination rates and purifying or directional selection (Villar et al. 2015; Lewis et al. 2016, 2021). We therefore assessed evolutionary turnover of accessible chromatin and protein coding exons to test whether the fusion of *Heliconius* chromosomes had a similar effect on functional loci as we observed at neutral sites. We used DNA sequence of CREs and gene exons from *D. iulia* to set a baseline for functional DNA turnover in the Heliconiini lineage. As expected, divergence time strongly affected conservation of both functional categories. We observed a similar degree of conservation between annotated *D. iulia* functional DNA and the *E. isabella* and *Heliconius* genomes, which represent 26 million years of sequence divergence (fig. 4*a*). Conservation continued to decrease with divergence time in comparisons against *M. cinxia* (65 million years diverged) and *Danaus plexippus* (85 million years diverged). For each species comparison, however, conservation of both exons and CREs displayed a strong correlation with chromosome length (Pearson’s *ρ* = 0.42–0.78). The correlation between functional DNA conservation and chromosome length decayed slightly with increased divergence time, most notably in exons, which showed a higher overall correlation (e.g., *ρ* = 0.78 for *H. melpomone* DNA) at lower divergence times. Thus, although some additional forces may affect functional DNA turnover, these results confirm that functional DNA elements remain subject to the same effects of recombination rate as neutral loci over macroevolutionary time.

To determine the extent to which chromosome fusions, and change in recombination rate, may have altered the turnover of functional DNA in *Heliconius*, we performed the same DNA conservation analysis using CREs and exons from *H. erato*. Testing the complete *H. erato* chromosomes allowed us to determine the effect of changes in recombination rate on functional DNA content over the past 11−19 million years postfusions. In contrast to our analysis of *D. iulia* functional DNA, conserved *H. erato* DNA showed a fairly strong correlation with chromosome length against the *H. melpomene* genome (Exons: *ρ* = 0.62, CREs: *ρ* = 0.75), but a substantial loss of association between chromosome length and DNA conservation between *H. erato* and more distantly related species with 31 chromosomes such as *E. isabella* (Exons: *ρ* = 0.26, CREs: *ρ* = 0.40).

We next examined the relationship between DNA sequence length and functional DNA turnover in the ancestral chromosome segments of the ten *H. erato* chromosomes that resulted from chromosome fusions. Functional DNA sequence conservation for these chromosome segments displayed a weaker correlation with sequence length against the *H. melpomene* assembly, but substantially greater correlation against the unfused heliconiine species and *M. cinxia* assemblies. In more diverged comparisons, correlation coefficients fell between the full *H. erato* chromosome values (supplementary fig. S20, Supplementary Material online, Pearson’s *ρ *= 0.41–0.74) and those from our analysis of *D. iulia* functional DNA. The effect size (slope of the relationship) of chromosome length on functional DNA turnover remained similar to that observed for the complete *H. erato* chromosomes. Together, these results suggest *Heliconius* may be at an intermediate stage where the evolutionary response of functional DNA turnover to changes in recombination rate is ongoing.

The reduced correlation between chromosome length and functional DNA conservation suggests that the relatively recent change in recombination rate in *Heliconius* may not have impacted functional DNA turnover to the same extent as stable recombination rates over a much longer period of time. To test this more explicitly, we performed multiple linear regression of *D. iulia* (fig. 4*c*) and *H. erato* (fig. 4*d*) functional elements to determine the effect of chromosome length relative to divergence time. Multiple regression models for both *D. iulia* and *H. erato* functional elements accounted for the substantial majority of variance in functional DNA conservation (*r*^2^ = 0.81–0.95). There was, however, a marked and significant (paired *t*-test, *P *<* *0.01) difference in the effect size of chromosome length between regression models of *D. iulia* and *H. erato* functional DNA. The impact of chromosome length and, by extension, recombination rate on DNA conservation was approximately seven times greater in *D. iulia* than for *H. erato*. The contrast between *D. iulia* and *H. erato* exon and CRE turnover thus suggests that recombination rate has a significant effect on DNA content putatively under selection, but that changes in recombination rate are likely more impactful on average rates of change across chromosomes over long periods of macroevolution than over shorter divergence times within genera. This impact may not be homogenous across the chromosome, however, and local changes in recombination rate may be important over microevolutionary timescales.

## Conclusions

Our analyses of four high-quality chromosome-level assemblies for *D. iulia*, *E. isabella, H. erato* and *H. melpomene* reveal important insights into the evolutionary impact of chromosome fusion. We have shown that Nymphalidae chromosomes differ significantly by size, both in terms of nucleotide composition and gene structure. Specifically, the ten short ancestral chromosomes had the highest repetitive element content, which possibly made them more likely to fuse with longer, more stable chromosomes, as occurred in the ancestor of *Heliconius*. This is likely a general property of small chromosomes, at least in Lepidoptera, as similar conclusions were drawn by comparative analyses of the *M. cinxia* genome (Ahola et al. 2014). Interestingly, both whole genomes and individual chromosomes with higher repetitive element content have longer introns on average, which appears to be driven by a higher density of repeats in introns compared with intergenic regions—a feature that, in theory, could also lead to the formation of new functional exons. We further show that the ten fusion events resulted in a dramatic change in the fate of the chromosomes involved, reducing the effective population size by increasing background selection. Finally, we explored whether chromosome fusions may have also changed rates of evolutionary turnover of accessible chromatin and protein coding exons and found that both exon and CRE conservation displayed a strong correlation with chromosome length, confirming that functional DNA elements are subject to the same effects of recombination rate over macroevolutionary time. These findings, and the genomic resources we provide, are not only important to understand how genomic architecture impacted lepidopteran evolution, but also to understand how the karyotype can affect species evolution at a broader biological scale.

## Materials and Methods

### DNA and RNA Extraction and Sequencing

Individuals of *E. isabella* were collected from partially inbred commercial stocks (Costa Rica Entomological Supplies, Alajuela, Costa Rica). High-quality, high-molecular-weight genomic DNA was extracted from late stages of pupae, dissecting up 100 mg of tissue, mainly thorax and wing imaginal disk, frozen in liquid nitrogen and homogenized in 9.2 ml buffer G2 (Qiagen Midi Prep Kit) adding 19 µl of RNAseA. The samples were then transferred in a 15 ml tube adding 0.2 µl of Protease K and incubated at ∼50 °C for 2 h. Samples were transferred to Genomic tip and processed with Qiagen Midi Prep Kit (Qiagen, Valencia, CA) following the manufacturer’s instructions. DNA was then precipitated using 2 ml 70% EtOH and dissolved in water.

From the same stocks, RNA was extracted separately from six adult tissue (four wings; three heads; four antennae, legs and mouth parts; thorax; abdomen 1–3, abdomen 4–6), and five tissue parts from early ommochrome stage of the pupae (head and mouth parts; wings, antennae and legs; thorax; abdomen 1–3, abdomen 4–6). Each tissue was frozen in liquid nitrogen and quickly homogenized in 500 µl Trizol, adding the remaining 500 µl Trizol at the end of the homogenization. Phase separation was performed adding 200 µl of cold chloroform. The upper phase was then transfer to RNeasy Mini spin column and processed with Qiagen RNeasy Mini Prep Kit (Qiagen, Valencia, CA) and DNAse purification using the Turbo DNA-free kit (Life Technologies, Carlsbad, CA) following the manufacturer’s instructions. All the extractions were finally pooled keeping the same final RNA concentration from all samples.

The Pacific Bioscience (PacBio) data were then sequenced at the Centre for Genomic Research, University of Liverpool using PacBio sequel SMRT cell (2.0 chemistry), whereas polyadenilated Illumina RNAseq data (125 bp x 2) where generated at the Institute of Applied Genomics (IGA), Udine, Italy.

### Genome Assembly

PacBio reads were corrected, trimmed and assembled using CANU v.1.8 + 356 changes (settings: genomeSize = 400 m; corMhapSensitivity = normal; corOutCoverage = 100; correctedErrorRate = 0.105; ovlMerThreshold = 500; batOptions = -dg 3 -db 3 -dr 1 -ca 500 -cp 50) (Koren et al. 2016). One problem with this type of long-read sequencing technology is the presence of postassembly insertions and deletions (indels) (Watson and Warr 2019). CANU can correct reads but does not remove all indels. The resulting raw assemblies were therefore corrected by remapping all uncorrected raw PacBio reads with pbmm2 v.1.0.0 and correcting the assembly with Arrow v.2.3.3 (https://github.com/PacificBiosciences/GenomicConsensus) for three iterations. To further error correct for indels we used short Illumina reads for *E. isabella* (Kozak et al. 2021) with Pilon v.1.23 (Walker et al. 2014) for five iterations. Assemblies were then processed with Purge Haplotigs v.20191008 (purge -a 85) (Roach et al. 2018) in order to remove haplocontigs, artificially duplicated genomic regions due to heterozygosity. To correct for mis-assemblies Polar Star (https://github.com/phasegenomics/polar_star) was employed. This algorithm calculates read depth of aligned PacBio reads to the assembly at each base. Read depth is then smoothed in a 100 bp sliding window, and regions of high, low, and normal depth are merged. Low read depth outliers are identified, and contigs are broken at each such location. Contigs were then rescaffolded using P_RNA_scaffolder (Zhu et al. 2018), which uses information from RNAseq mapping, and LRScaf v.1.1.5 (Qin et al. 2018), which uses information from long-reads. The resulting gaps were then filled using PacBio reads applying LR_Gapcloser v.1.1 (Xu et al. 2019). After the introduction of this new PacBio information, we repeated the previous polishing procedure using five iterations of Pilon, plus three more iterations with Illumina RNA-seq data to correct indels only. Before the chromosome-level scaffolding, we used synteny maps implemented with BLAST (Camacho et al. 2009) and ALLMAPS (Tang et al. 2015) to identify duplicated regions at the end of scaffolds, trimming them away.

Illumina RAD-seq data from Davey et al. (2016) were downloaded and aligned to the scaffolds of *E. isabella* assembly using STAR v.2.7.2c (Dobin et al. 2013) to generate the linkage map for the final scaffolding step. Duplicate reads were removed using the Picard v.2.0.1 MarkDuplicates tool (http://broadinstitute.github.io/picard). Indels were subsequently realigned using the RealignerTargetCreator and IndelRealigner tools from GATK v.3.8-1-0-gf15c1c3ef (Depristo et al. 2011). Individual SNPs were called using mpileup in SAMtools (Li et al. 2009) in combination with pileupParser2 and pileup2posterior modules from Lep-MAP v.3 (Rastas 2017). SNPs were filtered using Lep-MAP Filtering2 and the final linkage generated using SeparateChromosomes2, JoinSingles2All and OrderMarkers2 (parameters: dataTolerance = 0.001; distortionLod = 1; lodLimit = 14; femaleTheta = 0; maleTheta = 0.05). The scaffolding stage was performed with Lep-Anchor (Rastas 2017).

### Transcriptome Annotation

RNA-seq raw read data were filtered using Trimmomatic v.0.39 (Bolger et al. 2014) (ILLUMINACLIP:$ILLUMINACLIP: 2:30:10; SLIDINGWINDOW: 5:10; MINLEN: 100). RNA-Seq data were used as training data input to ab initio and de novo predictors and for direct alignments, and were mapped to the genome using STAR v.2.7.2c aligner (alignIntronMax = 500,000; alignSJoverhangMin = 10). The resultant sorted BAM files were used as training for the BRAKER v.2.1.5 pipeline (Hoff et al. 2016), which combines GeneMark-ES Suite v.4.30 (Lomsadze et al. 2005) and AUGUSTUS v.3.3.3 (Stanke et al. 2008), along with the masked genomes, generated with RepeatMasker v.4.1.0 (Smit et al. 2013) using the Lepidoptera database and protein alignments from closely related, or model species (*Drosophila melanogaster*, *B. mori, Bicyclus anynana*, *D. plexippus*, *H. erato*, and *H. melpomene*) using Exonerate v.2.4.0 (Slater and Birney 2005). BRAKER is an automated pipeline to predict genes that uses iterative training of AUGUSTUS to generate initial gene predictions. GeneMark-predicted genes are filtered and provided for AUGUSTUS training, followed by AUGUSTUS prediction, integrating the RNA-Seq and protein alignment information, to generate the final gene predictions.

To generate the de novo transcriptome assemblies, quality filtered reads assembled using Trinity v.2.10.0 (Grabherr et al. 2011) and generated transcript were subsequently aligned to the genome using Minimap2 v.2.17-r974-dirty (Li 2018). To generate the ab initio transcriptomes, reads were realigned to the genome using STAR with the same parameters with Braker predicted splice sites, and assembled using Stringtie v.2.1.3b (Pertea et al. 2015) and Cufflinks v.2.2.1 (Trapnell et al. 2010, 2012) for each sample. The assemblies derived from all samples within each program were merged using Stringtie –merge. All such generated transcriptomes (Trinity, BRAKER, StringTie, and Cufflinks) were merged used with STAR to remap once again all raw reads in order to evaluate all detectable splice-sites with Portcullis v.1.1.2 (Mapleson et al. 2018). Transcripts from ab initio and de novo assemblies (Trinity, StringTie, and Cufflinks) without supported splice-junctions were therefore filtered out, and only transcripts with unique intron chain were retained from all annotations.

The best putative protein-coding sequences were finally inferred using TransDecoder v5.5.0 (http://transdecoder.github.io/) (minimum amino acid length > 50) using homologs from UniProt database (Bateman 2019) and Lepidoptera proteome (see below) found with deltaBLAST v.2.7.1+ (Boratyn et al. 2012); and PFam domains (El-Gebali et al. 2019) with HMMscan v.3.3 (Eddy 1998) (e < 1e^−10^). For transcripts without a putative protein-coding region CPC2 (Kang et al. 2017) was adopted to identify putative noncoding transcripts.

### Chromosome Analysis

Chromosome mapping was carried out using orthologous genes between *D. iulia, E. Isabella, H. erato and H. melpomene* to define the level of gene conservation and translocations among chromosomes. Synteny maps were implemented with BLAST and ALLMAPS using Minimap2 to map *H. erato* and *H. melpomene* loci to the *D. iulia* and *E. isabell*a assemblies. This let us identify the putative junctions between the two fused chromosome pairs. The midpoints between the flanking orthologs of different homologous chromosomes were used to split the fused chromosomes in *H. erato* and *H. melpomene* in two, recreating the ancestral chromosome set. Chromosome mappings are illustrated using Circos (Krzywinski et al. 2009).

We explored the scaling relationship between chromosome size and repetitive elements across species and chromosome types (NFC, LFC and SFC) using SMATR v.3.4-8 (Warton et al. 2012), together with a Wilcoxon−Mann−Whitney rank sum test as implemented in the R function WILCOX.TEST (http://www.r-project.org) v.3 to test for differences in the relationships between chromosome size, GC content, and repetitive element content among chromosome types (chromosomes that were fused/unfused in *Heliconius*). In both cases, we adopted a stringent *P*-value threshold of 0.005 (Benjamin et al. 2017). We also calculated GC, repetitive element and coding-sequence (CDS) content within nonoverlapping 500 kb sliding windows for the chromosomes of all the species, with the order and orientation of the chromosomes determined based on synteny to *H. melpomene*. We used the annotation and an in house python script to extract strand specific intronic regions (BED format), whereas to extract strand specific intergenic regions the initial annotation was split into plus and minus strands and BEDTools complement v.2.29.0 (Quinlan and Hall 2010) used to generate intronic and intergenic regions. For both annotations BEDTools getfasta were used to extract their relative sequences analyzed with RepeatMasker. Their relative scaling coefficients and intercepts were subsequently analyzed with SMATR as reported above.

### Analysis of Genetic Diversity

To investigate whether chromosome fusions are associated with long-term shifts in levels of genetic diversity, we analyzed genome resequencing data from three to six additional individuals of each species (see supplementary table S1, Supplementary Material online for sample details and accession numbers). For newly sequenced individuals, DNA extraction was performed using the Qiagen DNeasy Blood and Tissue kit and libraries were prepared following a low-input Nextera-based DNA library prep protocol (skim sequencing) at the Cornell Institute of Technology Core Facility. Libraries were sequenced on a HiSeq4000. Reads were aligned to their respective reference assemblies using BWA MEM version 0.7.17 (Li 2013). Processing and sorting of SAM and BAM files was performed using SAMtools version 1.9 (Li et al. 2009), and duplicate reads were removed using Picard MarkDuplicates version 2.21.1 (https://broadinstitute.github.io/picard/). For genotyping we targeted only coding sites, as we were specifically interested in comparing levels of diversity among 4-fold degenerate third codon positions, which provide the most reliable comparison of relative diversity at nearly neutrally evolving sites in the genome. We therefore first defined the set of nonoverlapping coding intervals in the genome based on the annotations and then used bcftools call version 1.9 (Li 2011) with the -m option (https://samtools.github.io/bcftools/call-m.pdf) to call genotypes for this subset of sites. Only individual genotypes with read depth >10 and genotype quality (GQ) >30 were considered, except in *D. iulia*, where lower coverage sequencing meant that these thresholds had to be decreased to 5 and 20, respectively.

Four-fold degenerate sites (“4D sites” hereafter) were defined as third codon positions at which a substitution to any other base would not alter the encoded amino acid. This condition was evaluated by considering not only the codon sequence in the reference assembly, but also any variants observed in the resequenced individuals, and codons with more than one valiant site were automatically discarded, resulting in the most conservative possible set of 4D sites. These steps were implemented using the script codingSiteTypes.py available at (https://github.com/simonhmartin/genomics_general). Nucleotide diversity was computed in nonoverlapping windows of 500 4D sites each, using the script popgenWindows.py (https://github.com/simonhmartin/genomics_general). In *D. iulia*, because three of the re-sequenced individuals were from a small Key Largo population that has reduced diversity, we instead used pairwise diversity between one Key Largo individual and individual used for genome assembly (Lewis et al. 2021).

### Analysis of Functional DNA Turnover between Species

To determine the extent to which changes in per base pair recombination rate may have alter functional DNA turnover between species, we tested for sequence conservation of cis-regulatory loci and gene exons in species with and without the ten chromosome fusion events. CREs were derived from ATAC-seq data for midpupal wing tissue from *H. erato* and *D. iulia* (Lewis and Reed 2019; Lewis et al. 2021) ATAC-seq data were processed as previously described (Lewis and Reed 2019) and peaks were called using F-seq (Boyle et al. 2008). To reduce discrepancies between genome assembly annotations, which may have different degrees of gene fragmentation, we used annotated exons from *H. erato* and *D. iulia* to test for gene coding elements (Lewis et al. 2021). A custom script was then used to perform a reciprocal best-hit BLAST search with an e-value threshold of e^−10^ on ATAC-seq peaks and gene exons to identify conserved functional DNA sequences. In this analysis, 30% conservation would indicate that 30% of the source genome elements tested were found by our BLAST search in the target genome assembly. Peak and exon sets were split into files for each of the 30 (*D. iulia*) and 20 (*H. erato*) chromosomes. These data sets were then BLASTed against the *H. erato lativitta* (Lewis et al. 2016), *H. melpomene* (Davey et al. 2016), *E. isabella, D. iulia, M. cinxia* (Ahola et al. 2014), and *D. plexippus* (Gu et al. 2019) genome assemblies. Regression analysis and calculation of correlation coefficients was performed using the Python Statsmodels package.

## Supplementary Material

Supplementary data are available at *Molecular Biology and Evolution* online.

## Supplementary Material

msab185_Supplementary_Data

## References

[msab185-B1] Ahola V, Lehtonen R, Somervuo P, Salmela L, Koskinen P, Rastas P, Välimäki N, Paulin L, Kvist J, Wahlberg N, et al 2014. The Glanville fritillary genome retains an ancient karyotype and reveals selective chromosomal fusions in Lepidoptera. Nat Commun. 5:1–9. 10.1038/ncomms5737 PMC4164777 25189940

[msab185-B2] Barton NH. 1995. Linkage and the limits to natural selection. Genetics 140(2):821–841. 7498757 10.1093/genetics/140.2.821 PMC1206655

[msab185-B3] Bateman A. 2019. UniProt: a worldwide hub of protein knowledge. Nucleic Acids Res. 47(D1):D506–D515. 30395287 10.1093/nar/gky1049 PMC6323992

[msab185-B4] Van Belleghem SM, Rastas P, Papanicolaou A, Martin SH, Arias CF, Supple MA, Hanly JJ, Mallet J, Lewis JJ, Hines HM, et al 2017. Complex modular architecture around a simple toolkit of wing pattern genes. Nat Ecol Evol. 1(3):52. 28523290 10.1038/s41559-016-0052 PMC5432014

[msab185-B5] Benjamin DJ, Berger JO, Johannesson M, Nosek BA, Wagenmakers E-J, Berk R, Bollen KA, Brembs B, Brown L, Camerer C, et al 2017. Redefine statistical significance. Nat Hum Behav. 2(1):6−10. 10.1038/s41562-017-0189-z 30980045

[msab185-B6] Bernardi G, Olofsson B, Filipski J, Zerial M, Salinas J, Cuny G, Meunier-Rotival M, Rodier F. 1985. The mosaic genome of warm-blooded vertebrates. Science 228(4702):953–958. 4001930 10.1126/science.4001930

[msab185-B7] Bolger AM, Lohse M, Usadel B. 2014. Trimmomatic: a flexible trimmer for Illumina sequence data. Bioinformatics 30(15):2114–2120. 24695404 10.1093/bioinformatics/btu170 PMC4103590

[msab185-B8] Boratyn GM, Schäffer AA, Agarwala R, Altschul SF, Lipman DJ, Madden TL. 2012. Domain enhanced lookup time accelerated BLAST. Biol Direct. 7:12. 22510480 10.1186/1745-6150-7-12 PMC3438057

[msab185-B9] Boyle AP, Guinney J, Crawford GE, Furey TS. 2008. F-Seq: a feature density estimator for high-throughput sequence tags. Bioinformatics 24(21):2537–2538. 18784119 10.1093/bioinformatics/btn480 PMC2732284

[msab185-B10] Camacho C, Coulouris G, Avagyan V, Ma N, Papadopoulos J, Bealer K, Madden TL. 2009. BLAST+: Architecture and applications. *BMC Bioinformatics* 10(1):421. 10.1186/1471-2105-10-421 PMC2803857 20003500

[msab185-B11] Campos JL, Charlesworth B. 2019. The effects on neutral variability of recurrent selective sweeps and background selection. Genetics 212(1):287–303. 30923166 10.1534/genetics.119.301951 PMC6499526

[msab185-B12] Charlesworth B. 1990. Mutation-selection balance and the evolutionary advantage of sex and recombination. Genet Res. 55(3):199–221. [CrossRef][10.1017/S0016672300025532] 2394378 10.1017/s0016672300025532

[msab185-B13] Concha C, Wallbank RWR, Hanly JJ, Fenner J, Livraghi L, Rivera ES, Paulo DF, Arias C, Vargas M, Sanjeev M, et al 2019. Interplay between developmental flexibility and determinism in the evolution of mimetic Heliconius wing patterns. Curr Biol. 29(23):3996−4009.e4. 31735676 10.1016/j.cub.2019.10.010

[msab185-B14] Corbett-Detig RB, Hartl DL, Sackton TB. 2015. Natural selection constrains neutral diversity across a wide range of species. PLoS Biol. 13(4):262−274. 10.1371/journal.pbio.1002112 PMC4393120 25859758

[msab185-B15] Cutter AD, Payseur BA. 2013. Genomic signatures of selection at linked sites: unifying the disparity among species. Nat Rev Genet. 14(4):e1002112. 10.1038/nrg3425 PMC4066956 23478346

[msab185-B17] Davey JW, Chouteau M, Barker SL, Maroja L, Baxter SW, Simpson F, Joron M, Mallet J, Dasmahapatra KK, Jiggins CD. 2016. Major improvements to the *Heliconius melpomene* genome assembly used to confirm 10 chromosome fusion events in 6 million years of butterfly evolution. G3 Genes Genomes Genet. 6:695–708. 10.1534/g3.115.023655 PMC4777131 26772750

[msab185-B18] Depristo MA, Banks E, Poplin R, Garimella KV, Maguire JR, Hartl C, Philippakis AA, Del Angel G, Rivas MA, Hanna M, et al 2011. A framework for variation discovery and genotyping using next-generation DNA sequencing data. Nat Genet. 43:491–498. 21478889 10.1038/ng.806 PMC3083463

[msab185-B19] Dobin A, Davis CA, Schlesinger F, Drenkow J, Zaleski C, Jha S, Batut P, Chaisson M, Gingeras TR. 2013. STAR: ultrafast universal RNA-seq aligner. Bioinformatics 29(1):15–21. 23104886 10.1093/bioinformatics/bts635 PMC3530905

[msab185-B20] Dobzhansky T, White MJD. 1977. Animal cytology and evolution. Cambridge: Cambridge University Press.

[msab185-B21] Eddy SR. 1998. Profile hidden Markov models. Bioinformatics 14(9):755–763. 9918945 10.1093/bioinformatics/14.9.755

[msab185-B22] Edelman NB, Frandsen PB, Miyagi M, Clavijo B, Davey J, Dikow RB, García-Accinelli G, Van Belleghem SM, Patterson N, Neafsey DE, et al 2019. Genomic architecture and introgression shape a butterfly radiation. Science 366(6465):594–599. 31672890 10.1126/science.aaw2090 PMC7197882

[msab185-B23] El-Gebali S, Mistry J, Bateman A, Eddy SR, Luciani A, Potter SC, Qureshi M, Richardson LJ, Salazar GA, Smart A, et al 2019. The Pfam protein families database in 2019. Nucleic Acids Res. 47(D1):D427–D432. 30357350 10.1093/nar/gky995 PMC6324024

[msab185-B24] Feulner PGD, De-Kayne R. 2017. Genome evolution, structural rearrangements and speciation. J Evol Biol. 30(8):1488–1490. 28786195 10.1111/jeb.13101

[msab185-B25] Fradin H, Kiontke K, Zegar C, Gutwein M, Lucas J, Kovtun M, Corcoran DL, Baugh LR, Fitch DHA, Piano F, et al 2017. Genome architecture and evolution of a unichromosomal asexual Nematode. Curr Biol. 27(19):2928–2939. e6. 28943090 10.1016/j.cub.2017.08.038 PMC5659720

[msab185-B26] Grabherr MG, Haas BJ, Yassour M, Levin JZ, Thompson D. A, Amit I, Adiconis X, Fan L, Raychowdhury R, Zeng Q, et al 2011. Full-length transcriptome assembly from RNA-Seq data without a reference genome. Nat Biotechnol. 29(7):644–652. 21572440 10.1038/nbt.1883 PMC3571712

[msab185-B27] Grbić M, Van Leeuwen T, Clark RM, Rombauts S, Rouzé P, Grbić V, Osborne EJ, Dermauw W, Ngoc PCT, Ortego F, et al 2011. The genome of *Tetranychus urticae* reveals herbivorous pest adaptations. Nature 479(7374):487–492. 22113690 10.1038/nature10640 PMC4856440

[msab185-B28] Gu L, Reilly PF, Lewis JJ, Reed RD, Andolfatto P, Walters JR. 2019. Dichotomy of dosage Compensation along the Neo Z chromosome of the Monarch butterfly. Curr Biol. 29(23):4071–4077.e3. 31735674 10.1016/j.cub.2019.09.056 PMC6901105

[msab185-B29] Guerrero RF, Kirkpatrick M. 2014. Local adaptation and the evolution of chromosome fusions. Evolution (N.Y.). 68(10):2747–2756. [CrossRef][10.1111/evo.12481] 10.1111/evo.12481 24964074

[msab185-B30] Haddrill PR, Halligan DL, Tomaras D, Charlesworth B. 2007. Reduced efficacy of selection in regions of the *Drosophila* genome that lack crossing over. Genome Biol. 8(2):R18. 17284312 10.1186/gb-2007-8-2-r18 PMC1852418

[msab185-B31] Hauffe HC, Searle JB. 1998. Chromosomal heterozygosity and fertility in house mice (*Mus musculus domesticus*) from Northern Italy. Genetics 150(3):1143–1154. 9799266 10.1093/genetics/150.3.1143 PMC1460399

[msab185-B32] Hill WG, Robertson A. 1966. The effect of linkage on limits to artificial selection. Genet Res. 8(3):269–294. [CrossRef][10.1017/S0016672300010156] 5980116

[msab185-B33] Hoff KJ, Lange S, Lomsadze A, Borodovsky M, Stanke M. 2016. BRAKER1: unsupervised RNA-Seq-based genome annotation with GeneMark-ET and AUGUSTUS. Bioinformatics 32(5):767–769. 26559507 10.1093/bioinformatics/btv661 PMC6078167

[msab185-B35] Kang YJ, Yang DC, Kong L, Hou M, Meng YQ, Wei L, Gao G. 2017. CPC2: a fast and accurate coding potential calculator based on sequence intrinsic features. Nucleic Acids Res. 45(W1):W12–W16. 28521017 10.1093/nar/gkx428 PMC5793834

[msab185-B36] Kent TV, Uzunović J, Wright SI. 2017. Coevolution between transposable elements and recombination. Philos Trans R Soc B Biol Sci. 372(1736):20160458. doi: 10.1098/rstb.2016.0458. 10.1098/rstb.2016.0458 PMC5698620 29109221

[msab185-B37] Kim S, Cho YS, Kim HM, Chung O, Kim H, Jho S, Seomun H, Kim J, Bang WY, Kim C, et al 2016. Comparison of carnivore, omnivore, and herbivore mammalian genomes with a new leopard assembly. Genome Biol. 17(1):1–12. 27802837 10.1186/s13059-016-1071-4 PMC5090899

[msab185-B38] Davey JW, Chouteau M, Barker SL, Maroja L, Baxter SW, Simpson F, Merrill RM, Joron M, Mallet J, Dasmahapatra KK, et al 2016. Major improvements to the *Heliconius melpomene* genome assembly used to confirm 10 chromosome fusion events in 6 million years of butterfly evolution. G3 (Bethesda) 6(3):695–708. 26772750 10.1534/g3.115.023655 PMC4777131

[msab185-B39] Koren S, Walenz BP, Berlin K, Miller JR, Bergman NH, Phillippy AM. 2016. Canu: scalable and accurate long-read assembly via adaptive k-mer weighting and repeat separation, 1–35. 10.1101/gr.215087.116 PMC5411767 28298431

[msab185-B40] Kozak KM, Joron M, McMillan WO, Jiggins CD. 2021. Rampant Genome-Wide Admixture across the Heliconius Radiation. *Genome Biol Evol*. 13(7):evab099. doi:10.1093/gbe/evab099. 10.1093/gbe/evab099 PMC8283734 33944917

[msab185-B41] Kozak KM, Wahlberg N, Neild AFE, Dasmahapatra KK, Mallet J, Jiggins CD. 2015. Multilocus species trees show the recent adaptive radiation of the mimetic heliconius butterflies. Syst Biol. 64(3):505–524. 25634098 10.1093/sysbio/syv007 PMC4395847

[msab185-B42] Krzywinski M, Schein J, Birol I, Connors J, Gascoyne R, Horsman D, Jones SJ, Marra MA. 2009. Circos: an information aesthetic for comparative genomics. Genome Res. 19(9):1639−1645. 19541911 10.1101/gr.092759.109 PMC2752132

[msab185-B43] Lewis JJ, Van Belleghem SM, Riccardo P, Danko CG, Reed RD. 2020. Many functionally connected loci foster adaptive diversification along a neotropical hybrid zone. Sci Adv. 6(39):eabb8617. 32978147 10.1126/sciadv.abb8617 PMC7518860

[msab185-B44] Lewis JJ, van der Burg KRL, Mazo-Vargas A, Reed RD. 2016. ChIP-Seq-annotated *Heliconius erato* genome highlights patterns of cis-regulatory evolution in Lepidoptera. Cell Rep. 16(11):2855–2863. 27626657 10.1016/j.celrep.2016.08.042

[msab185-B45] Lewis JJ, Cicconardi F, Martin SH, Reed RD, Danko CG, Montgomery SH. 2021. The Dryas iulia genome supports multiple gains of a W chromosome from a B chromosome in butterflies. *Genome Biol Evol*. 13(7):evab128. doi: 10.1093/gbe/evab128. 10.1093/gbe/evab128 PMC8290107 34117762

[msab185-B46] Lewis JJ, Geltman RC, Pollak PC, Rondem KE, van Belleghem SM, Hubisz MJ, Munn PR, Zhang L, Benson C, Mazo-Vargas A, et al 2019. Parallel evolution of ancient, pleiotropic enhancers underlies butterfly wing pattern mimicry. Proc Natl Acad Sci USA. 116(48):24174−24183. 31712408 10.1073/pnas.1907068116 PMC6883815

[msab185-B47] Lewis JJ, Reed RD. 2019. Genome-wide regulatory adaptation shapes population-level genomic landscapes in *Heliconius*. Mol Biol Evol. 36(1):159−173. 30452724 10.1093/molbev/msy209 PMC6340471

[msab185-B48] Li H. 2011. A statistical framework for SNP calling, mutation discovery, association mapping and population genetical parameter estimation from sequencing data. Bioinformatics 27(21):2987–2993. 21903627 10.1093/bioinformatics/btr509 PMC3198575

[msab185-B49] Li H. 2013. Aligning sequence reads, clone sequences and assembly contigs with BWA-MEM. 00:1–3. Available from: https://arxiv.org/abs/1303.3997.

[msab185-B50] Li H. 2018. Minimap2: pairwise alignment for nucleotide sequences. Bioinformatics 34(18):3094–3100. 29750242 10.1093/bioinformatics/bty191 PMC6137996

[msab185-B51] Li H, Handsaker B, Wysoker A, Fennell T, Ruan J, Homer N, Marth G, Abecasis G, Durbin R, 1000 Genome Project Data Processing Subgroup. 2009. The sequence alignment/map format and SAMtools. Bioinformatics 25(16):2078–2079. 19505943 10.1093/bioinformatics/btp352 PMC2723002

[msab185-B52] Lomsadze A, Ter-Hovhannisyan V, Chernoff YO, Borodovsky M. 2005. Gene identification in novel eukaryotic genomes by self-training algorithm. Nucleic Acids Res. 33(20):6494–6506. 16314312 10.1093/nar/gki937 PMC1298918

[msab185-B53] Lunt DH, Kumar S, Koutsovoulos G, Blaxter ML. 2014. The complex hybrid origins of the root knot nematodes revealed through comparative genomics. PeerJ. 2:e356. 24860695 10.7717/peerj.356 PMC4017819

[msab185-B54] MacKay TFC, Richards S, Stone EA, Barbadilla A, Ayroles JF, Zhu D, Casillas S, Han Y, Magwire MM, Cridland JM, et al 2012. The *Drosophila melanogaster* genetic reference panel. Nature 482(7384):173–178. 22318601 10.1038/nature10811 PMC3683990

[msab185-B55] Mackintosh A, Laetsch DR, Hayward A, Charlesworth B, Waterfall M, Vila R, Lohse K. 2019. The determinants of genetic diversity in butterflies. Nat Commun. 10(1):3466. 31371715 10.1038/s41467-019-11308-4 PMC6672018

[msab185-B56] Mandrioli M, Manicardi GC. 2020. Holocentric chromosomes. PLoS Genet. 16(7):e1008918. 32730246 10.1371/journal.pgen.1008918 PMC7392213

[msab185-B57] Mapleson D, Venturini L, Kaithakottil G, Swarbreck D. 2018. Efficient and accurate detection of splice junctions from RNA-seq with Portcullis. Gigascience 7(12):1–11. 10.1093/gigascience/giy131 PMC6302956 30418570

[msab185-B58] Martin SH, Davey JW, Salazar C, Jiggins CD. 2019. Recombination rate variation shapes barriers to introgression across butterfly genomes. PLoS Biol. 17(2):e2006288. 30730876 10.1371/journal.pbio.2006288 PMC6366726

[msab185-B59] Martin SH, Möst M, Palmer WJ, Salazar C, McMillan WO, Jiggins FM, Jiggins CD. 2016. Natural selection and genetic diversity in the butterfly *Heliconius melpomene*. Genetics 203(1):525–541. 27017626 10.1534/genetics.115.183285 PMC4858797

[msab185-B60] Moest M, Van Belleghem SM, James JE, Salazar C, Martin SH, Barker SL, Moreira GRP, Merot C, Joron M, Nadeau NJ, et al 2020. Selective sweeps on novel and introgressed variation shape mimicry loci in a butterfly adaptive radiation. PLoS Biol. 18(2):e3000597. 32027643 10.1371/journal.pbio.3000597 PMC7029882

[msab185-B61] Pertea M, Pertea GM, Antonescu CM, Chang TC, Mendell JT, Salzberg SL. 2015. StringTie enables improved reconstruction of a transcriptome from RNA-seq reads. Nat Biotechnol. 33(3):290–295. 25690850 10.1038/nbt.3122 PMC4643835

[msab185-B62] Presgraves DC. 2005. Recombination enhances protein adaptation in *Drosophila melanogaster*. Curr Biol. 15(18):1651–1656. 16169487 10.1016/j.cub.2005.07.065

[msab185-B63] Qin M, Wu S, Li A, Zhao F, Feng H, Ding L, Chang Y, Ruan J. 2018. LRScaf: improving draft genomes using long noisy reads. *bioRxiv* [Internet], 374868. Available from: https://www.biorxiv.org/content/early/2018/07/24/374868?%3Fcollection= 10.1186/s12864-019-6337-2 PMC6902338 31818249

[msab185-B64] Qu Z, Nong W, So WL, Barton-Owen T, Li Y, Leung TCN, Li C, Baril T, Wong AYP, Swale T, et al 2020. Millipede genomes reveal unique adaptations during myriapod evolution. PLoS Biol. 18(9):e3000636. 32991578 10.1371/journal.pbio.3000636 PMC7523956

[msab185-B65] Quinlan AR, Hall IM. 2010. The BEDTools manual. Genome.

[msab185-B66] Rastas P. 2017. Lep-MAP3: robust linkage mapping even for low-coverage whole genome sequencing data. Bioinformatics 33(23):3726–3732. 29036272 10.1093/bioinformatics/btx494

[msab185-B67] Ray DA, Grimshaw JR, Halsey MK, Korstian JM, Osmanski AB, Sullivan KAM, Wolf KA, Reddy H, Foley N, Stevens RD, et al 2019. Simultaneous TE analysis of 19 Heliconiine butterflies yields novel insights into rapid TE-based genome diversification and multiple SINE births and deaths. Genome Biol Evol. 11(8):2162–2177. 31214686 10.1093/gbe/evz125 PMC6685494

[msab185-B68] Roach MJ, Schmidt SA, Borneman AR. 2018. Purge Haplotigs: synteny reduction for third-gen diploid genome assemblies. *bioRxiv* [Internet], 286252. Available from: https://www.biorxiv.org/content/early/2018/03/22/286252.full.pdf+html 10.1186/s12859-018-2485-7 PMC6267036 30497373

[msab185-B69] Schmitz J, Brosius J. 2011. Exonization of transposed elements: a challenge and opportunity for evolution. Biochimie 93(11):1928–1934. 21787833 10.1016/j.biochi.2011.07.014

[msab185-B70] Sela N, Kim E, Ast G. 2010. The role of transposable elements in the evolution of non-mammalian vertebrates and invertebrates. Genome Biol. 11(6):R59. [CrossRef][10.1186/gb-2010-11-6-r59][PMC][20525173] 20525173 10.1186/gb-2010-11-6-r59 PMC2911107

[msab185-B71] Slater GSC, Birney E. 2005. Automated generation of heuristics for biological sequence comparison. BMC Bioinformatics 6(1):31–11. 15713233 10.1186/1471-2105-6-31 PMC553969

[msab185-B72] Smit A, Hubley R, Green P. 2013. RepeatMasker Open-4.0. 2013-2015. Available from: http://www.repeatmasker.org.

[msab185-B73] Stanke M, Diekhans M, Baertsch R, Haussler D. 2008. Using native and syntenically mapped cDNA alignments to improve de novo gene finding. Bioinformatics 24(5):637–644. 18218656 10.1093/bioinformatics/btn013

[msab185-B74] De Storme N, Mason A. 2014. Plant speciation through chromosome instability and ploidy change: cellular mechanisms, molecular factors and evolutionary relevance. Curr Plant Biol. 1:10–33.

[msab185-B75] Tang H, Zhang X, Miao C, Zhang J, Ming R, Schnable JC, Schnable PS, Lyons E, Lu J. 2015. ALLMAPS: robust scaffold ordering based on multiple maps. Genome Biol. 16(1):3. 25583564 10.1186/s13059-014-0573-1 PMC4305236

[msab185-B76] Trapnell C, Roberts A, Goff L, Pertea G, Kim D, Kelley DR, Pimentel H, Salzberg SL, Rinn JL, Pachter L. 2012. Differential gene and transcript expression analysis of RNA-seq experiments with TopHat and Cufflinks. Nat Protoc. 7(3):562–578. 22383036 10.1038/nprot.2012.016 PMC3334321

[msab185-B77] Trapnell C, Williams BA, Pertea G, Mortazavi A, Kwan G, Van Baren MJ, Salzberg SL, Wold BJ, Pachter L. 2010. Transcript assembly and quantification by RNA-Seq reveals unannotated transcripts and isoform switching during cell differentiation. Nat Biotechnol. 28(5):511–515. 20436464 10.1038/nbt.1621 PMC3146043

[msab185-B78] Veller C, Edelman NB, Muralidhar P, Nowak MA. 2020. Variation in genetic relatedness is determined by the aggregate recombination process. Genetics. 216(4):985–994. 33109528 10.1534/genetics.120.303680 PMC7768252

[msab185-B79] Veller C, Kleckner N, Nowak MA. 2019. A rigorous measure of genome-wide genetic shuffling that takes into account crossover positions and Mendel’s second law. Proc Natl Acad Sci USA. 116(5):1659−1668. 30635424 10.1073/pnas.1817482116 PMC6358705

[msab185-B80] Villar D, Berthelot C, Aldridge S, Rayner TF, Lukk M, Pignatelli M, Park TJ, Deaville R, Erichsen JT, Jasinska AJ, et al 2015. Enhancer evolution across 20 mammalian species. Cell 160(3):554–566. 25635462 10.1016/j.cell.2015.01.006 PMC4313353

[msab185-B81] de Vos JM, Augustijnen H, Bätscher L, Lucek K. 2020. Speciation through chromosomal fusion and fission in Lepidoptera. Philos Trans R Soc Lond B Biol Sci. 375(1806):20190539. 32654638 10.1098/rstb.2019.0539 PMC7423279

[msab185-B82] Walker BJ, Abeel T, Shea T, Priest M, Abouelliel A, Sakthikumar S, Cuomo CA, Zeng Q, Wortman J, Young SK, et al 2014. Pilon: an integrated tool for comprehensive microbial variant detection and genome assembly improvement. PLoS One 9(11):e112963. 25409509 10.1371/journal.pone.0112963 PMC4237348

[msab185-B83] Warton DI, Duursma RA, Falster DS, Taskinen S. 2012. smatr 3- an R package for estimation and inference about allometric lines. Methods Ecol Evol. 3:257−259.

[msab185-B84] Watson M, Warr A. 2019. Errors in long-read assemblies can critically affect protein prediction. Nat Biotechnol. 37:127–128. 30670796 10.1038/s41587-018-0004-z

[msab185-B85] Xu GC, Xu TJ, Zhu R, Zhang Y, Li SQ, Wang HW, Li JT. 2019. LR-Gapcloser: a tiling path-based gap closer that uses long reads to complete genome assembly. Gigascience 8(1):1–14. 10.1093/gigascience/giy157 PMC6324547 30576505

[msab185-B86] Zhu BH, Xiao J, Xue W, Xu GC, Sun MY, Li JT. 2018. P_RNA_scaffolder: a fast and accurate genome scaffolder using paired-end RNA-sequencing reads. BMC Genomics 19(1):1–13. 29499650 10.1186/s12864-018-4567-3 PMC5834899

