# Supplementary material for: Chromosome Fusion Affects Genetic Diversity and Evolutionary Turnover of Functional Loci but Consistently Depends on Chromosome Size
Source: Mol Biol Evol. 2021 Jun 19;38(10):4449–62. doi: 10.1093/molbev/msab185 (PMC8476138; doi:10.1093/molbev/msab185)
Supplement: msab185_Supplementary_Data [file msab185_supplementary_data.zip › SupplementaryFigures.MBE.pdf]

Chromosome fusion affects genetic diversity and evolutionary turnover of functional loci, but consistently depends on chromosome size

Francesco Cicconardi[1,2,a,*], James J Lewis[3,4,b,*], Simon H Martin[5], Robert D. Reed[4], Charles G Danko[3], Stephen H Montgomery[1,c]

Supplementary Figures

Affiliations: [1]School of Biological Sciences, University of Bristol Bristol - Life Sciences Building, Bristol, UK; [2]Department of Zoology, University of Cambridge, Downing Street, Cambridge, CB2 3EJ; [3]Baker Institute for Animal Health, Cornell University, Ithaca, NY; [4]Ecology and Evolutionary Biology, Cornell University, Ithaca, NY; [5]Institute of Evolutionary Biology, University of Edinburgh, Edinburgh, UK.

Correspondence: [a]f.cicconardi@bristol.ac.uk; [b] jjl336@cornell.edu; [c]s.montgomery@bristol.ac.uk
*contributed equally

Figure S1. Genome assembly and transcriptome workflows ____ pg 2
Figure S2. Depth of coverage and overall GC distribution ____ pg 3
Figure S3. N(x) statistics distribution____ pg 3
Figure S4. Error correction of the assembled genome ____ pg 4
Figure S5. Insecta_db9 BUSCO gene statistics ____ pg 5
Figure S6. Sequence-level synteny between D. iulia, E. isabella and H. melpomene ____ pg 6
Figure S7. Homologous not-fused chromosome comparisons ____ pg 7
Figure S8. Homologous fused chromosome comparisons ____ pg 8
Figure S9. Gene feature distributions ____ pg 9
Figure S10. Repeat landscape plots ____ pg 10
Figure S11. Repeat composition across chromosome ____ pg 11
Figure S12. Repetitive elements vs chromosome lengths ____ pg 12
Figure S13. Genomic feature correlations ____ pg 12
Figure S14. Intron size distributions ____ pg 13
Figure S15. SMART intronic vs intergenic repeats ____ pg 14
Figure S16. Intronic repeats in SFC ____ pg 15
Figure S17. Chromosome physical length vs map length ____ pg 16
Figure S18. Relationship between chromosome length and nucleotide diversity ____ pg 17

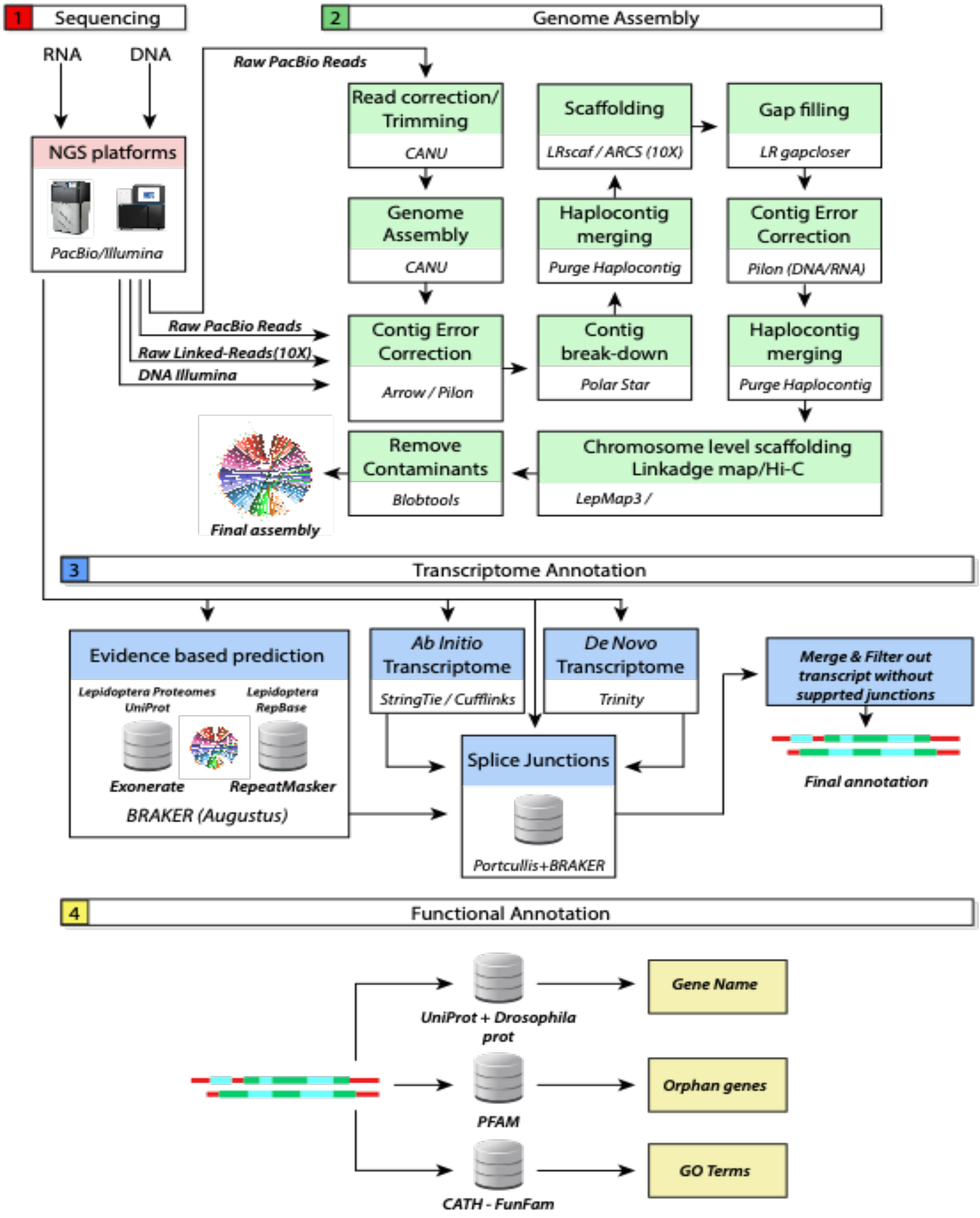

Figure S1. Genome assembly and transcriptome workflows. The different sequenced data sets (1) are used for the assembly workflow (2), and the transcriptomic annotation (3) of the *E. isabella* genome assembly. The resulted annotated sequences were then used as a query to search for homologies and its relative GO term (4).

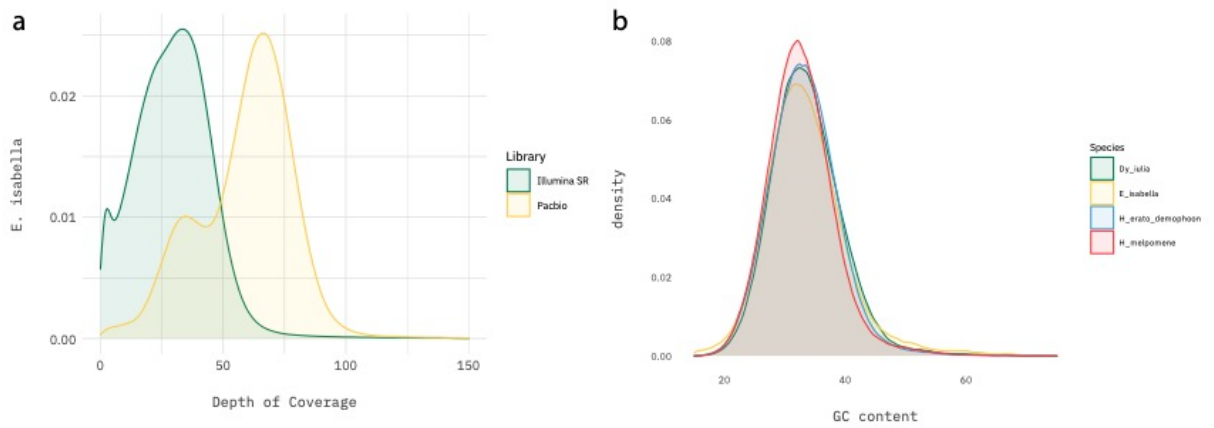

Figure S2. Depth of coverage and overall GC distribution. a) Depth of coverage for the short-read Illunmina and PacBio libraries. c) The GC distribution of *E. Isabella* with *D. iulia, H. erato* and *H. melpomene* showing very high similarities.

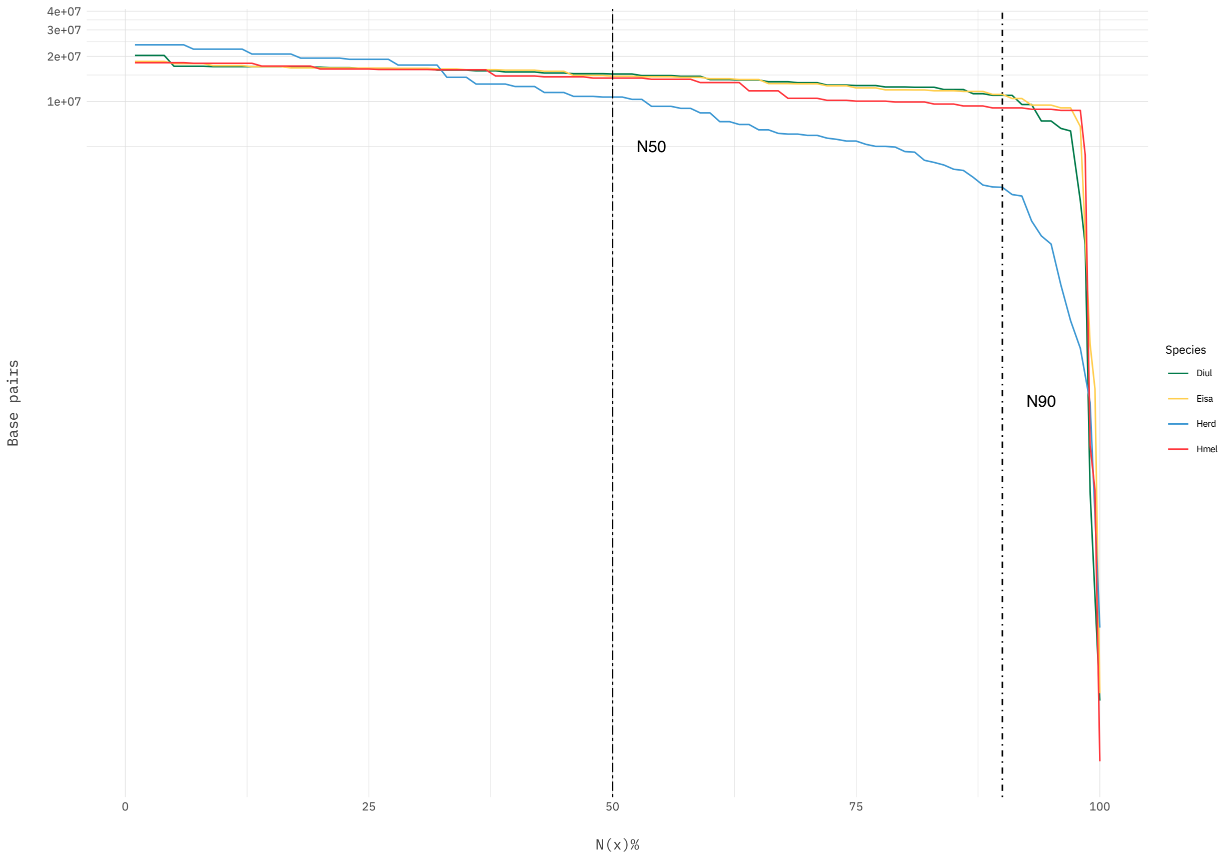

Figure S3. *N(x)* statistics distribution. The distribution of N(x) distribution for the chromosole-level genome of *E. isabella* compared with the ones of *D. iulia, H. erato* and *H. melpomene*. Our assembled genomes show a comparable if not better contiguity than the other genome assemblies.

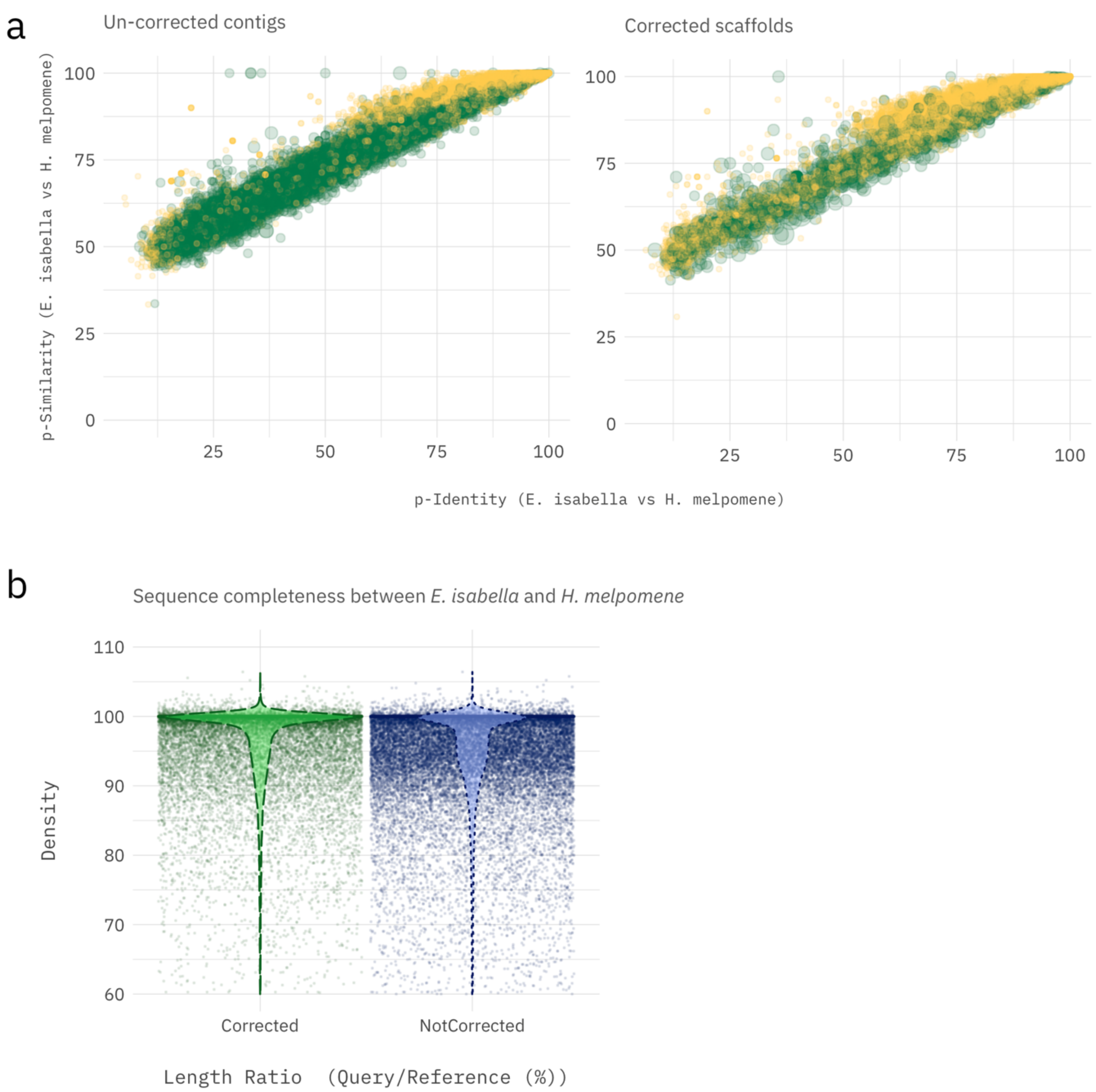

Figure S4. Error correction of the assembled genome. a) Scatter plot of *H. melpomene* proteome mapped using exonerate to the *E. Isabella* uncorrected contigs and corrected scaffolds. Each circle represents a mapped protein, in yellow genes with no indel, in green genes with indel, their sizes are proportional to the number of indel for that specific gene. The two plots clearly show the reduced number of genes with reported indels, showing successful error corrections. Also, the remaining green dots have very low *p*-identity and *p*-similarity, therefore they could represent unspecific alignment of pseudo-exons. b) Violin plots showing the length distributions between the mapped genes on *E. Isabella* against its reference in *H. melpomene*, in uncorrected contigs and corrected scaffolds. It shows how the distribution in after the error correction is more picked around 100%, and comparably lower around 95%, underlining an effective error correction.

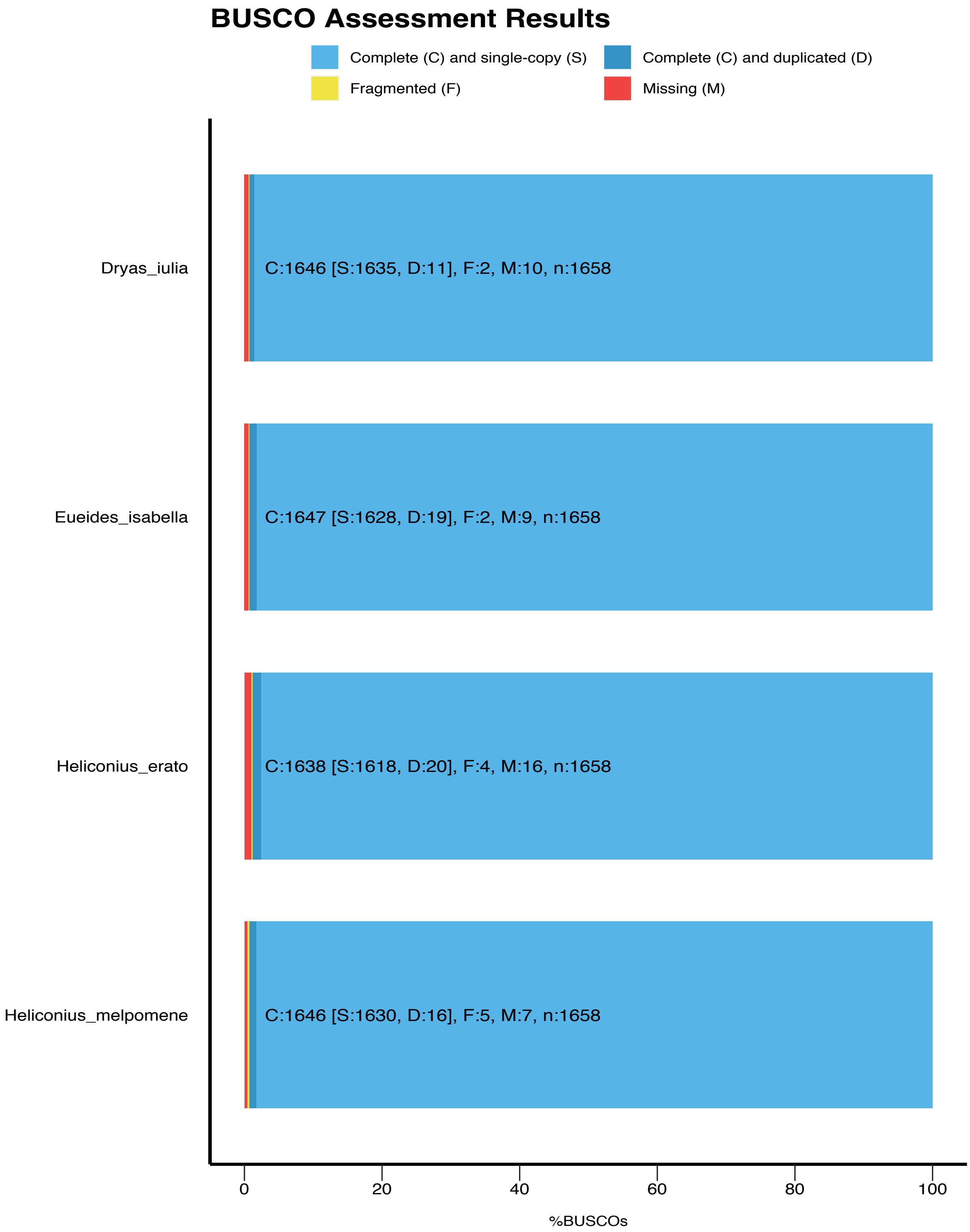

Figure S5. Insecta_db9 BUSCO gene statistics. Stacked histogram of Universal Single-Copy Orthologs (BUSCO) for E. Isabella genome and the other reference genomes. All species show a very similar and good completeness.

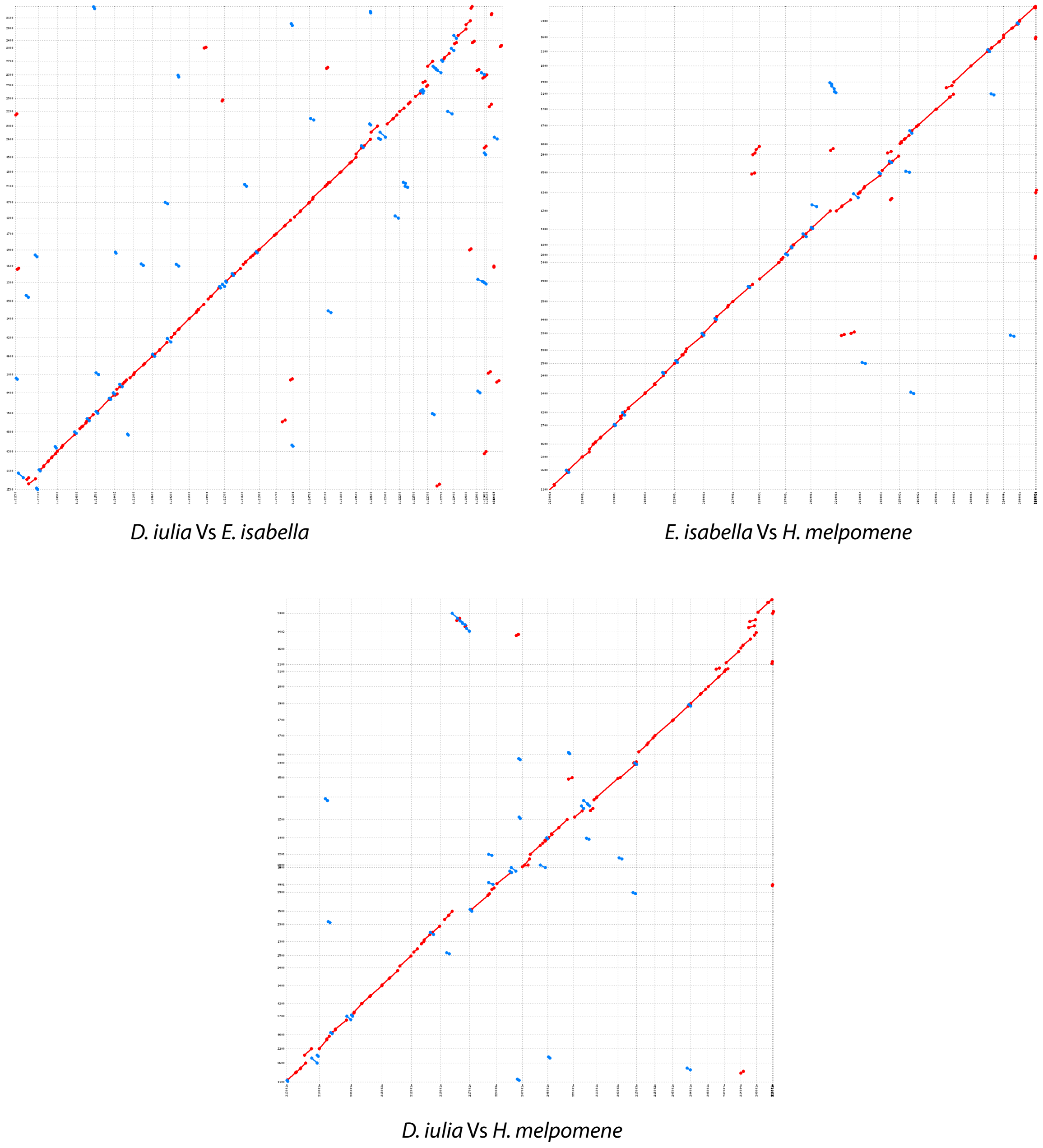

Figure S6. Sequence-level synteny between *D. iulia*, *E. isabella* and *H. melpomene*. Dotplot of all genomic pairwise positions between Chromosomes from *D. iulia* vs *E. isabella*, *D. iulia* vs *H. melpomene*, and *E. isabella* vs *H. melpomene*, using a window of 500kb and a minimum *p*-identity of 0.70. The red and blue represent the forward and reverse complemented hits, respectively.

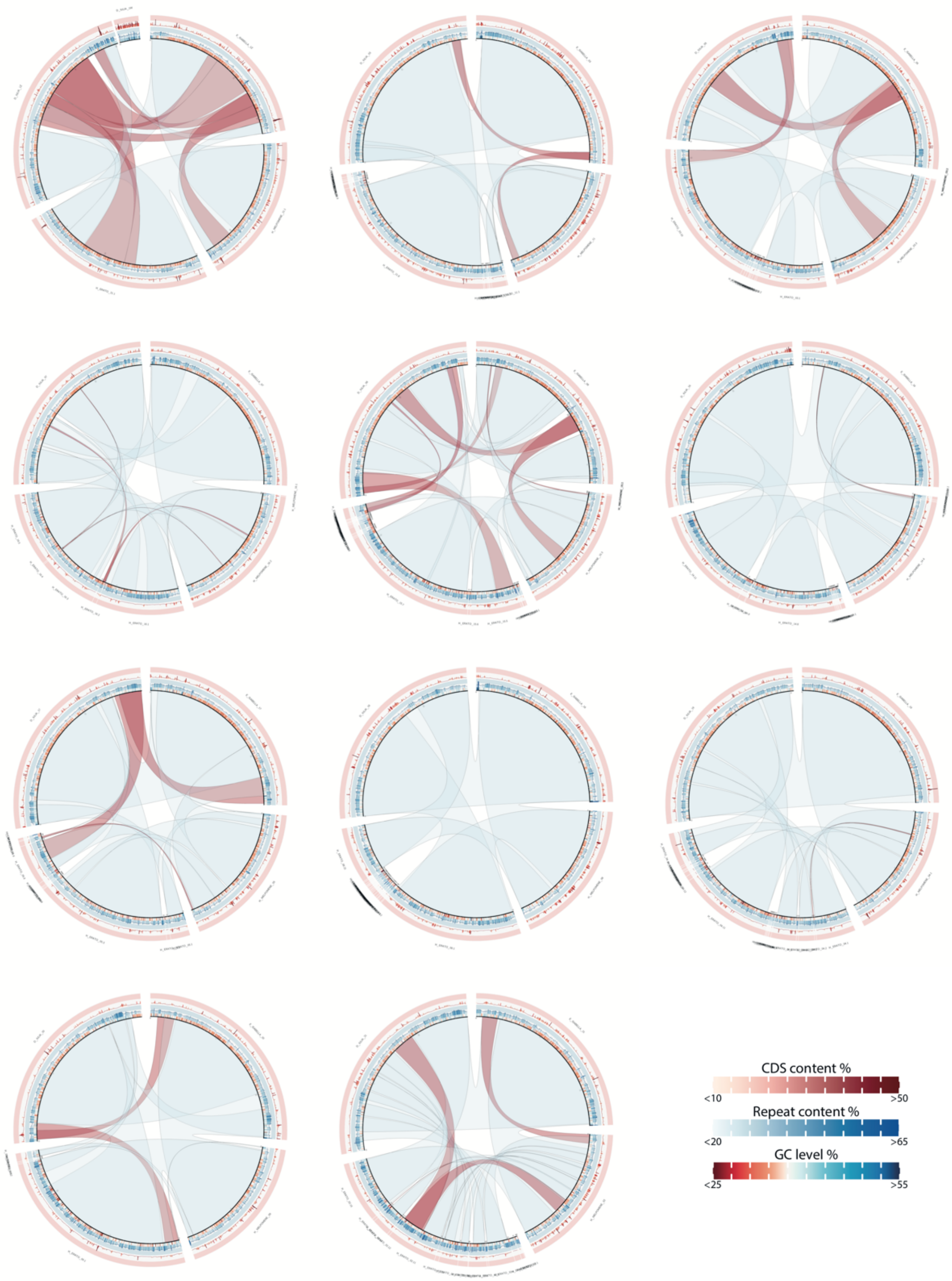

Figure S7. Homologous not-fused chromosome comparisons. Synteny plots for not-fused chromosomes. The comparisons are shown only for the flanking species, in order to avoid confusion. For layer of for the plots indicate: 1) Coding region content, higher density is shown in darker red; 2) Repetitive element richness, higher density is shown in darker blue; 3) GC level, in red AT-rich regions while in blue more GC-rich content; 4) Ribbons showing synteny blocks (light blue), blocks are red coloured when the block is inverted in the comparison. The ideogram scale is in Mbp.

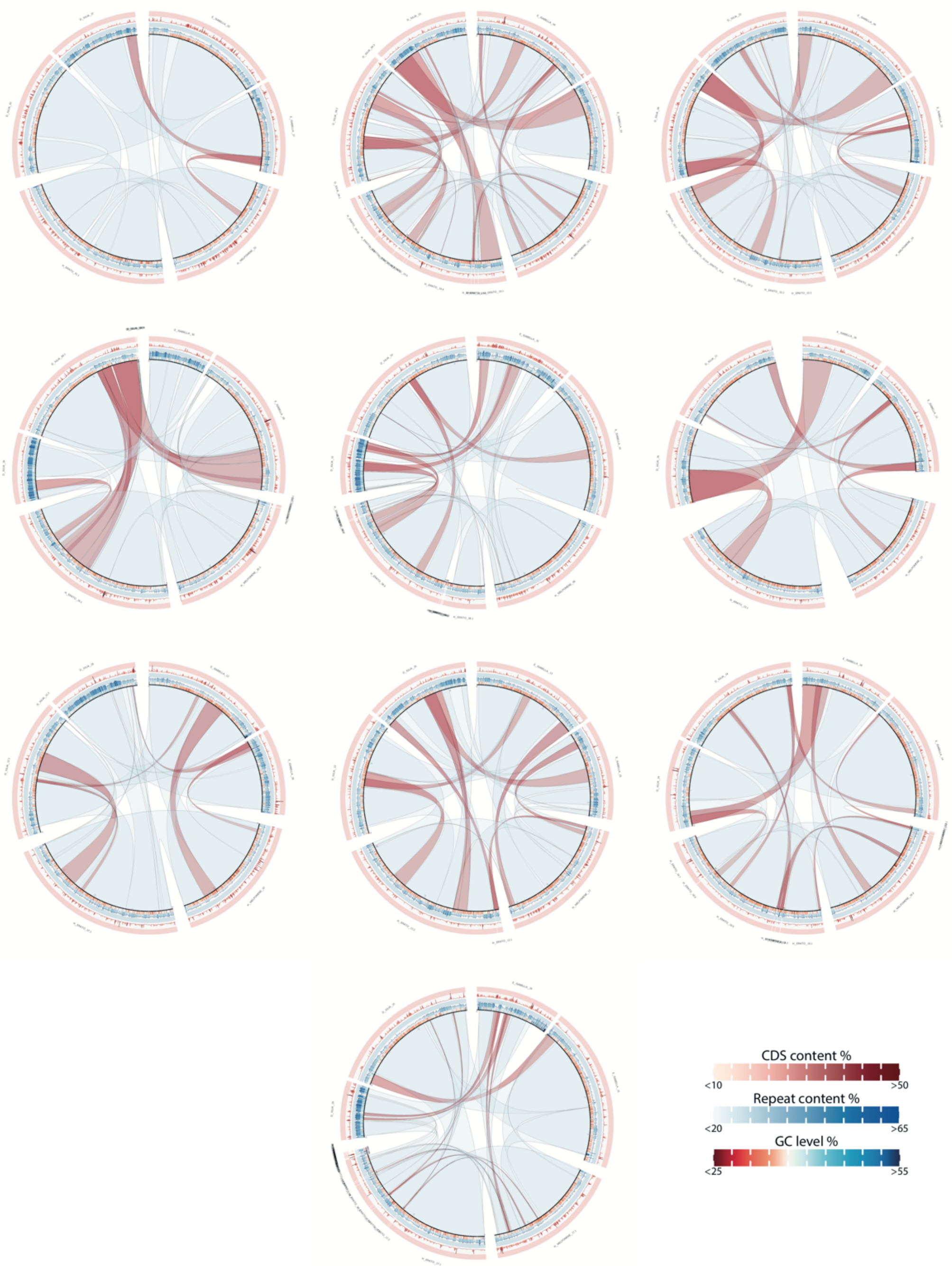

Figure S8. Homologous fused chromosome comparisons. Synteny plots for chromosomes fused in *Heliconius* together with their homologous chromosomes in *D. iulia* and *E. isabella*. The comparisons are shown only for the flanking species, in order to avoid confusion. For layer of for the plots indicate: 1) Coding region content, higher density is shown in darker red; 2) Repetitive element richness, higher density is shown in darker blue; 3) GC level, in red AT-rich regions while in blue more GC-rich content; 4) Ribbons showing synteny blocks (light blue), blocks are red colored when the block is inverted in the comparison. The ideogram scale is in Mbp.

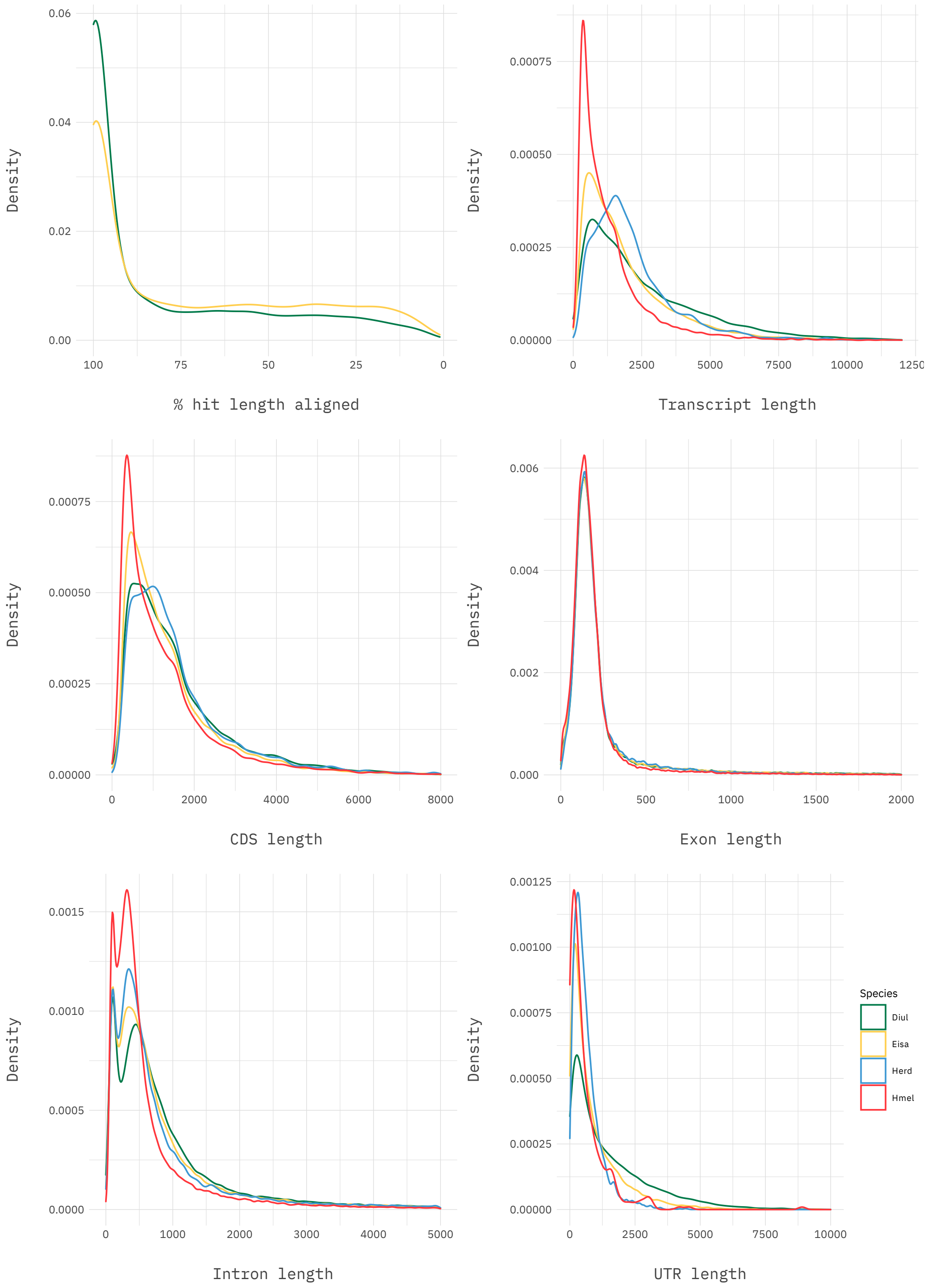

Figure S9. Gene feature distributions. Distribution of CDS length percentage compared with its best BLAST hit for *E. Isabella* and *D. iulia*. Length distributions for general features of the final E. Isabella trascriptome with the other annotated reference genomes.

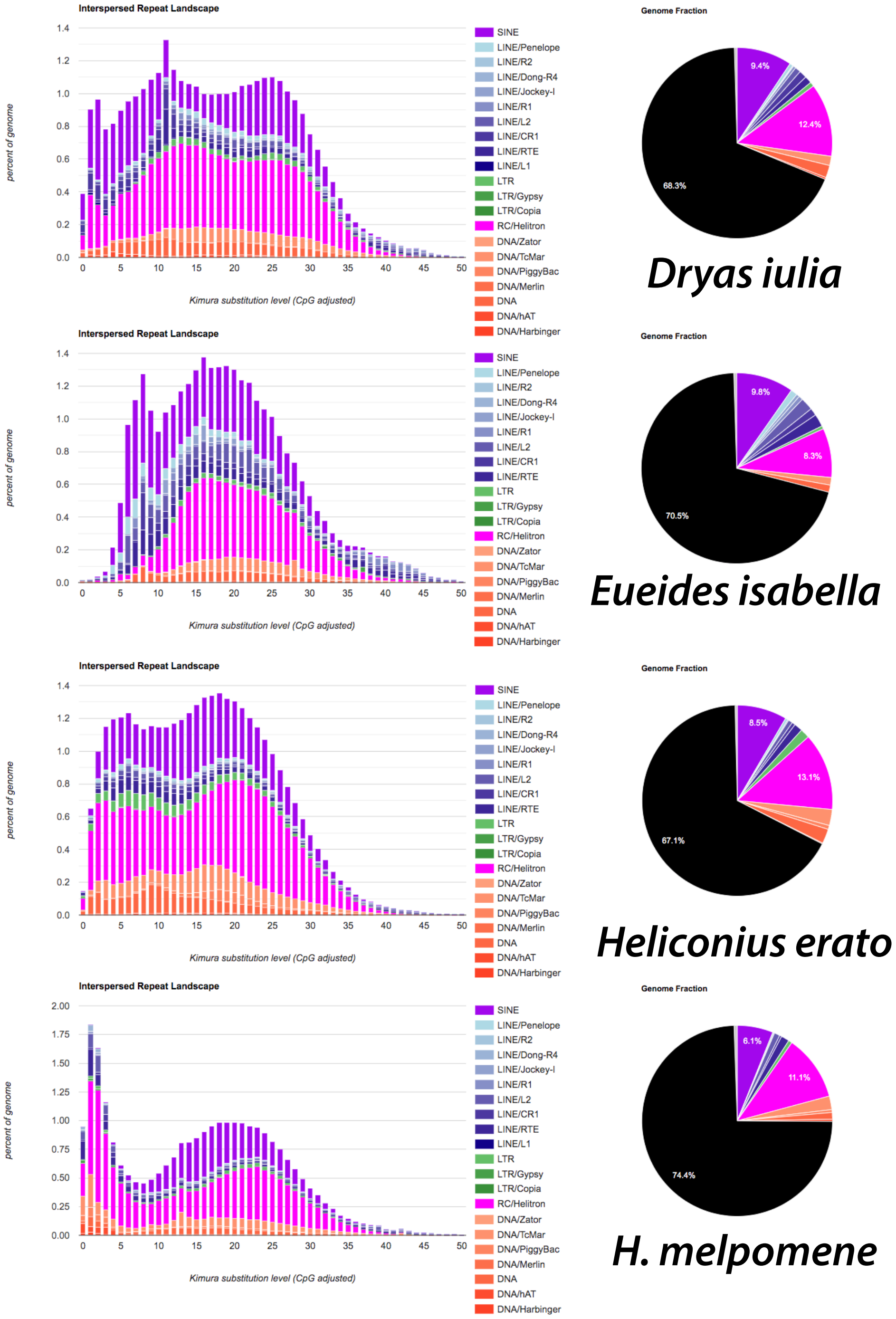

Figure S10. Repeat landscape plots. Stacked histogram of repetitive elements distributed according to their kimura distances (*x*-axis). On their side pie charts showing the percentage of repeat typology for the entire assembly. In *E. isabella* there is a depletion of young repeats ($K < 5$), while in H. melpomene this fraction is significantly increased, compared with older repeats that seem to be significantly depleted.

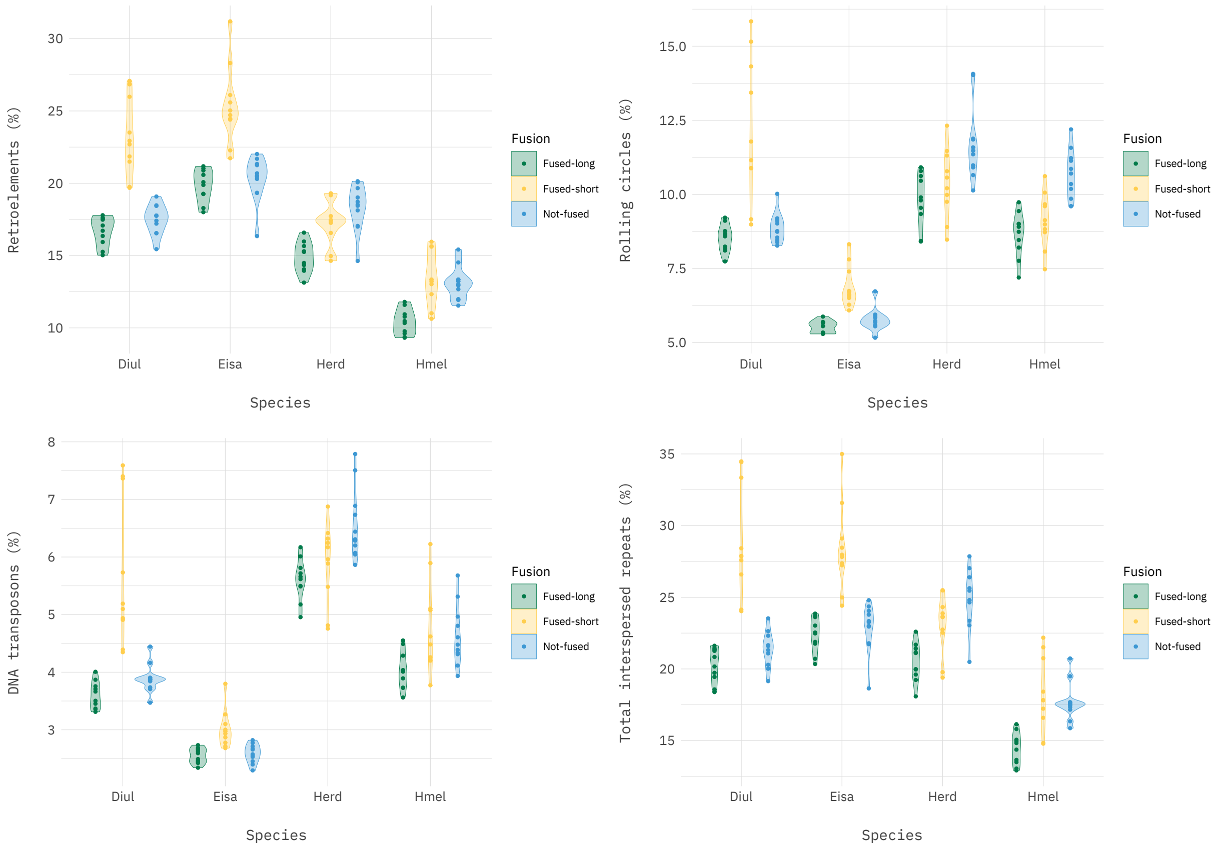

Figure S11. Repeat composition across chromosome. Violin plots showing the different repetitive element typologies among chromosomes. In *Dryas iulia* and *E. isabella* the short-fused chromosomes (SFCs) seem to have a higher proportion for all the four typologies.

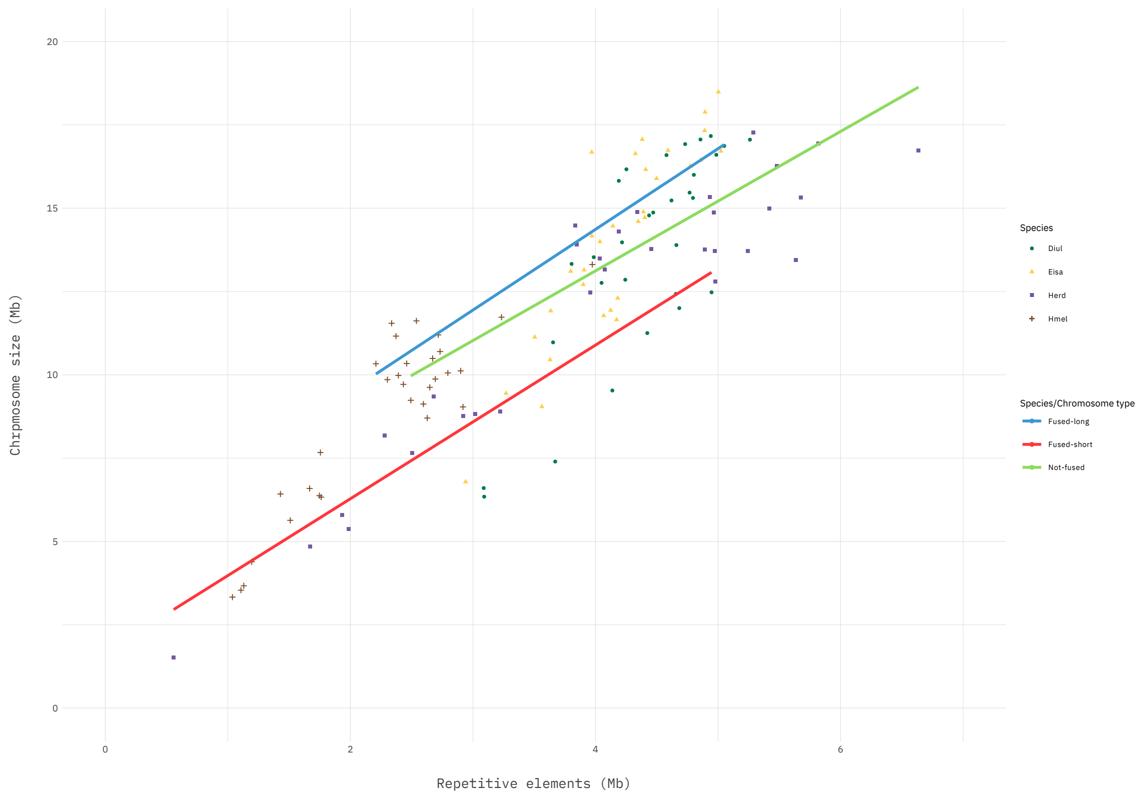

Figure S12. Repetitive elements vs chromosome lengths. Log–log plots of scaling between the total repetitive elements and its corresponding chromosome. A significant grade shifts exists between fused-short chromosomes and long and not fused chromosomes across all species ($P < 0.00001$).

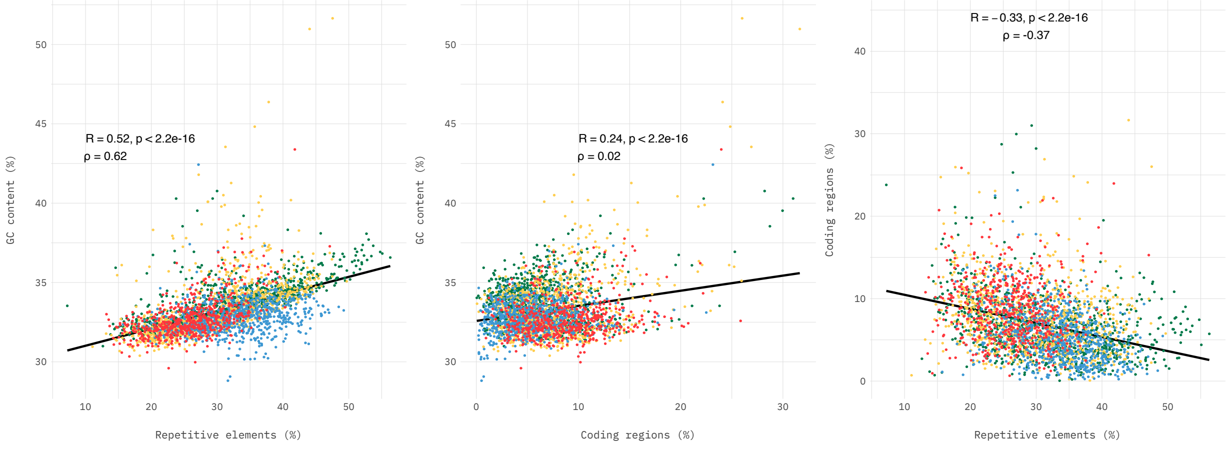

Figure S13. Genomic feature correlations. Scatter plots showing possible correlation between GC, repetitive elements and coding regions in a not overlapping 100kb window, across the genome of the four species (colours). There seems to be a stronger correlation only between CG content and repeats, and only mildly between CDS and repeats, this is probably due to introns being proportionally more affected by transposable element compared with intergenic regions.

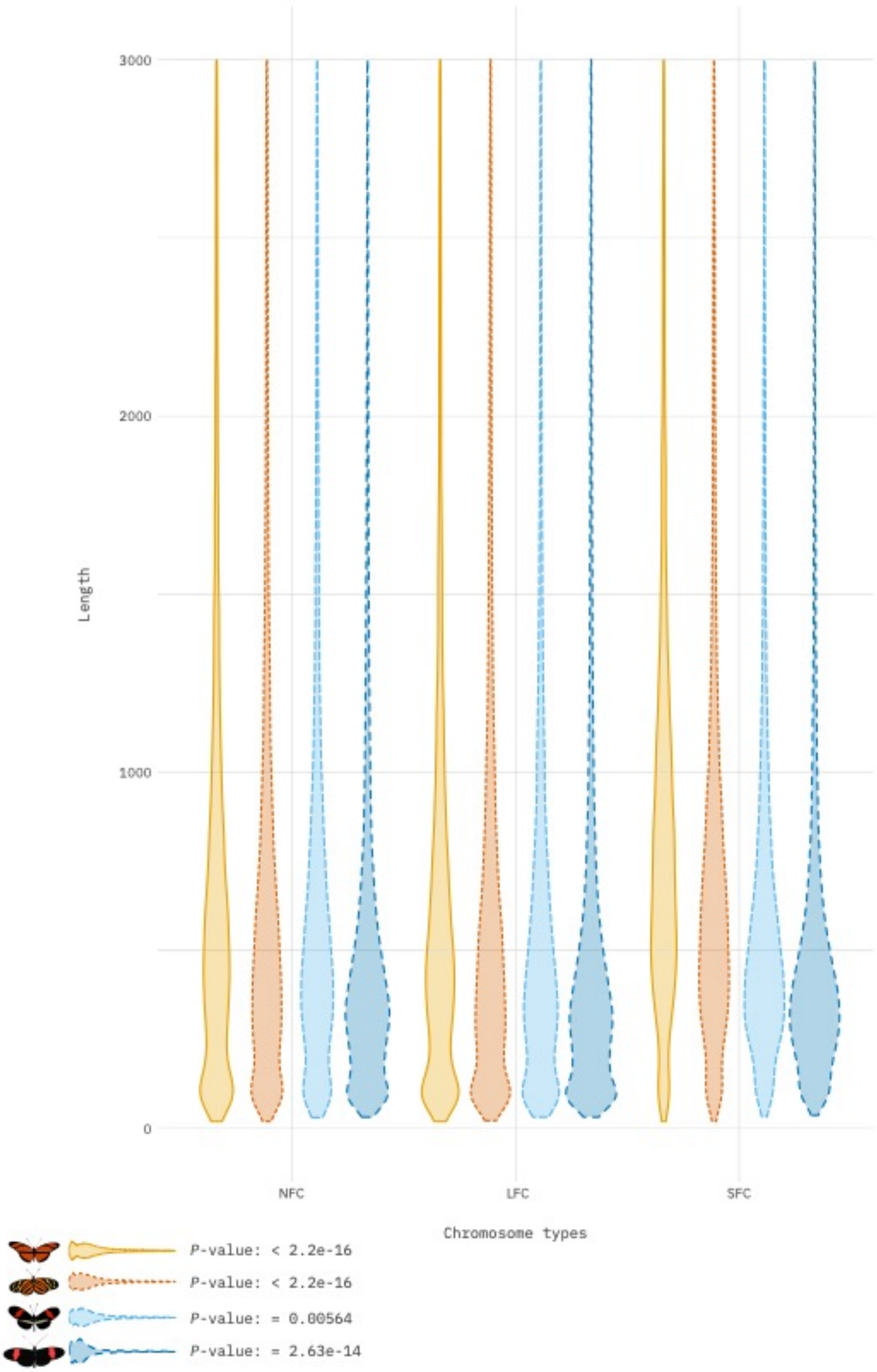

Figure S14. Intron size distributions. Violin plots of intron lengths for the different species in the three chromosome types. Short introns seem to be depleted in short-fused chromosomes (SFCs), compared with not fused (NFCs) and long-fused (LFCs) chromosomes.

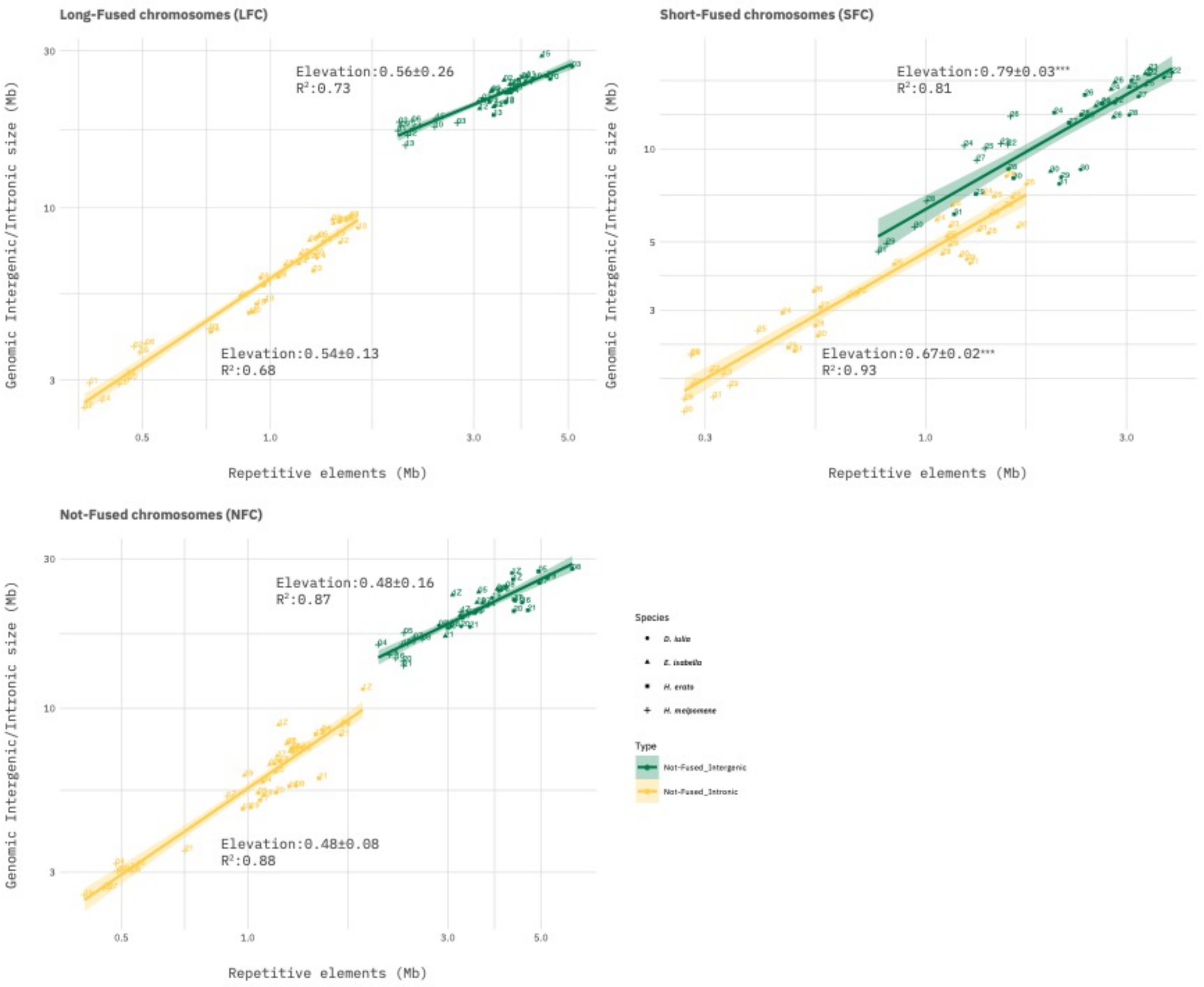

Figure S15. SMART intronic vs intergenic repeats. Log–log plots of scaling between repeats and intergenic/intronic regions for the three chromosome types. The elevation between intronic and intergenic regions is significant difference only in short-fused chromosomes ($P = 6.7 \times 10^{-10}$) (Table S3).

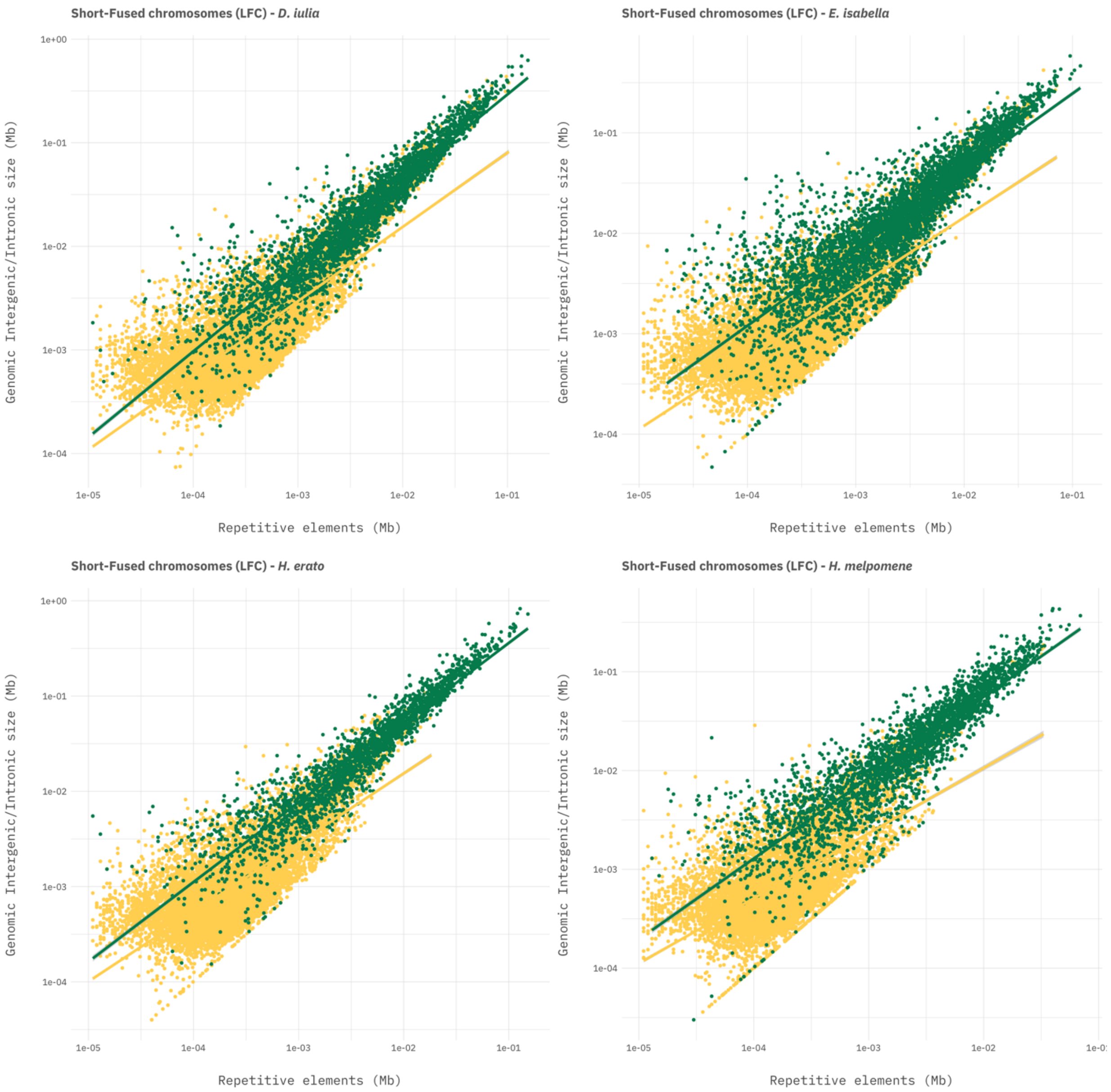

Figure S16. Intronic repeats in SFC. Log–log plots of scaling between repeats and intergenic/intronic regions for short-fused chromosomes for the four species. The elevation between intronic and intergenic regions is significant difference only in *D. iulia* and *E. isabella* (P = 2.22×10^{-16}) (Table S4).

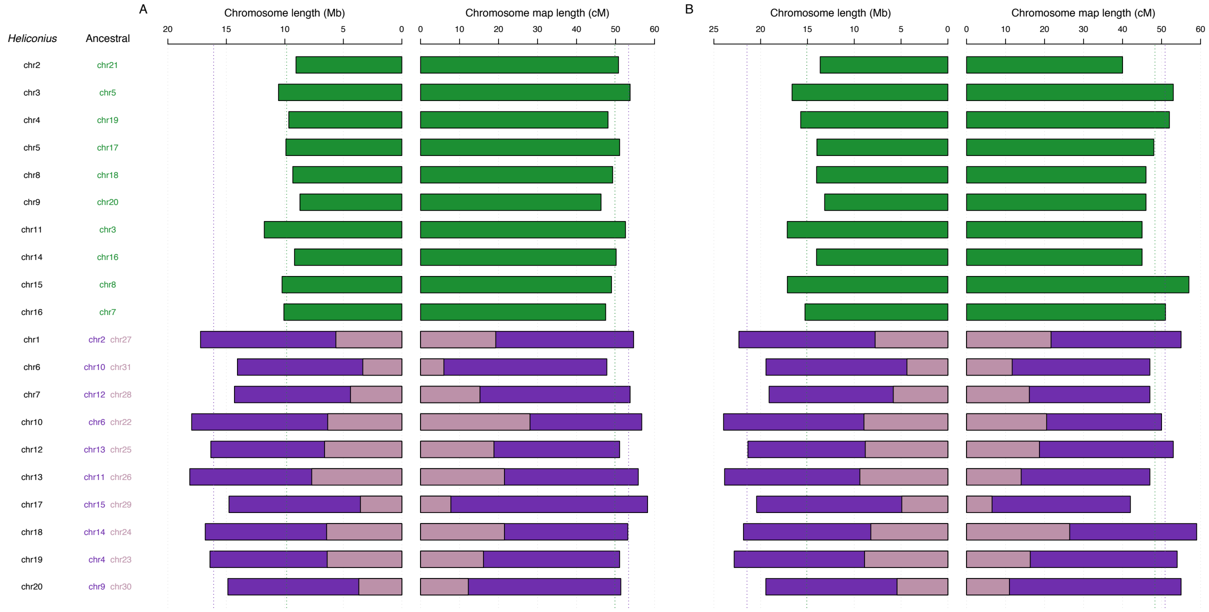

Figure S17. Chromosome physical length vs map length. Physical lengths (bars to the left) and linkage map lengths (bars to the right) are shown for *H. melpomene* (a) and *H. erato* (b). Chromosome numbers are given on the far left, along with the ancestral chromosomes they correspond to. The three groups of ancestral chromosomes are coloured as in other figures: green = not-fused chromosomes (NFCs), purple = long-fused chromosomes (LFCs), pink = short-fused chromosomes (SFCs). Average physical and map lengths for both NFCs and LFCs+SFCs (i.e.: fused *Heliconius* chromosomes) are shown by vertical dashed lines. While physical lengths for fused chromosomes are much larger than unfused cromosomes, their linkage map lengths are similar, indicating a reduction in recombination rate per bp.

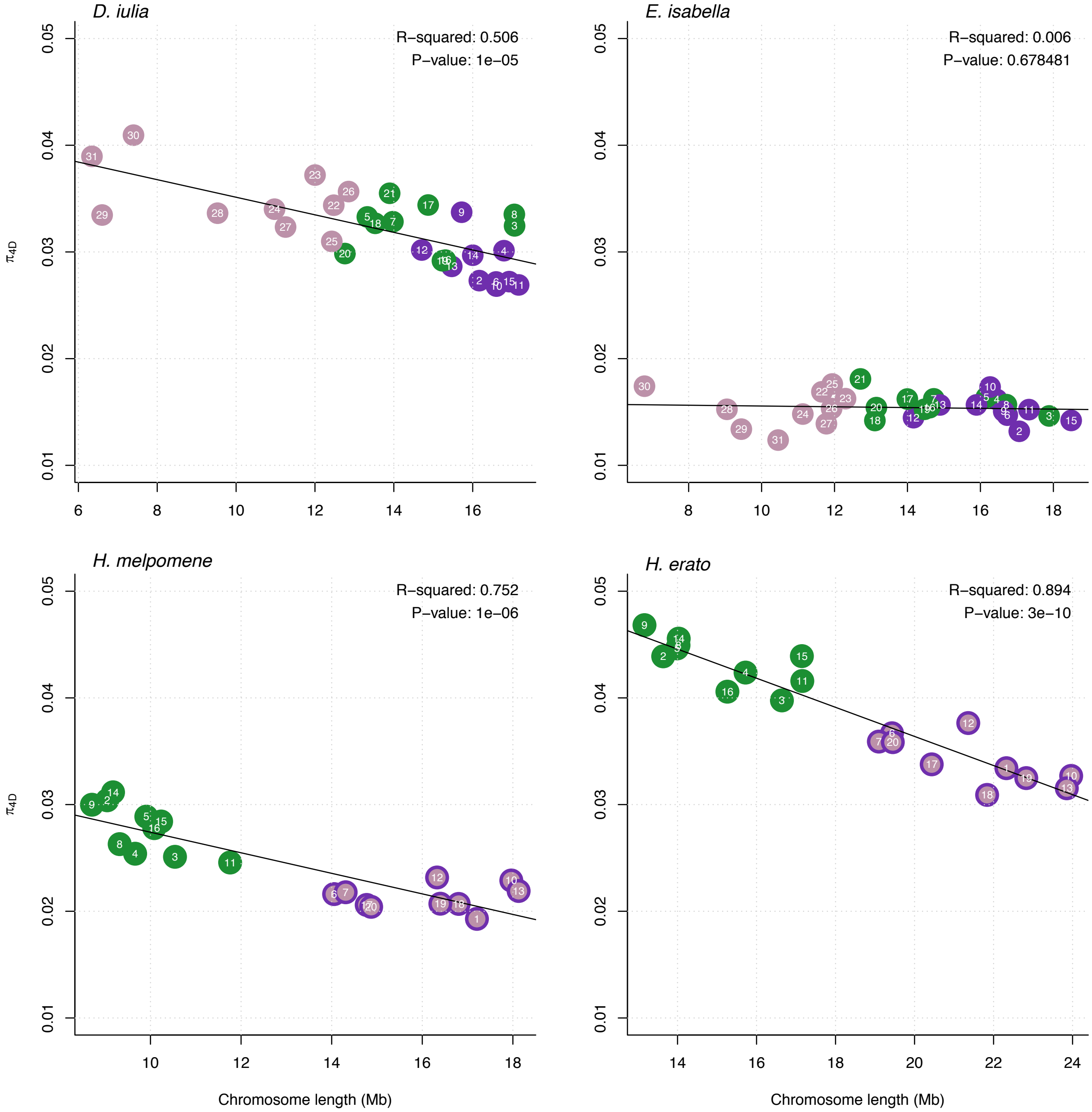

Figure S18. Relationship between chromosome length and nucleotide diversity. For each of the four study species, nucleotide diversity at four-fold degenerate codon positions (π_{4D}) averaged across each chromosome is plotted against chromosome physical length. A negative relationship between silent site diversity and chromosome length is indicative of lower effective population size on longer chromosomes, as expected if lower recombination rates lead to stronger background selection and/or genetic hitchhiking. Points are coloured as in other figures: green = not-fused chromosomes (NFCs), purple = long-fused chromosomes (LFCs), pink = short-fused chromosomes (SFCs). Combined pink/purple points in *Heliconius* indicates fused chromosomes. A fitted linear regression line is shown, along with associated r^2 and p-values.
